# Liver sinusoidal endothelial cells show reduced scavenger function and downregulation of Fc gamma receptor IIb, yet maintain a preserved fenestration in the *Glmp*^*gt/gt*^ mouse model of slowly progressing liver fibrosis

**DOI:** 10.1371/journal.pone.0293526

**Published:** 2023-11-01

**Authors:** Milton Boaheng Antwi, Gianina Dumitriu, Jaione Simón-Santamaria, Javier Sánchez Romano, Ruomei Li, Bård Smedsrød, Anders Vik, Winnie Eskild, Karen Kristine Sørensen

**Affiliations:** 1 Department of Medical Biology, UiT-The Arctic University of Norway, Tromsø, Norway; 2 Section of Haematology, University Hospital of North Norway, Tromsø, Norway; 3 Department of Clinical Medicine, UiT-The Arctic University of Norway, Tromsø, Norway; 4 Department of Biosciences, University of Oslo, Oslo, Norway; University of Melbourne (Peter Doherty Institute for Infection and Immunity), AUSTRALIA

## Abstract

Liver sinusoidal endothelial cells (LSECs) are fenestrated endothelial cells with a unique, high endocytic clearance capacity for blood-borne waste macromolecules and colloids. This LSEC scavenger function has been insufficiently characterized in liver disease. The *Glmp*^*gt/gt*^ mouse lacks expression of a subunit of the MFSD1/GLMP lysosomal membrane protein transporter complex, is born normal, but soon develops chronic, mild hepatocyte injury, leading to slowly progressing periportal liver fibrosis, and splenomegaly. This study examined how LSEC scavenger function and morphology are affected in the *Glmp*^*gt/gt*^ model. FITC-labelled formaldehyde-treated serum albumin (FITC-FSA), a model ligand for LSEC scavenger receptors was administered intravenously into *Glmp*^*gt/gt*^ mice, aged 4 months (peak of liver inflammation), 9–10 month, and age-matched *Glmp*^*wt/wt*^ mice. Organs were harvested for light and electron microscopy, quantitative image analysis of ligand uptake, collagen accumulation, LSEC ultrastructure, and endocytosis receptor expression (also examined by qPCR and western blot). In both age groups, the *Glmp*^*gt/gt*^ mice showed multifocal liver injury and fibrosis. The uptake of FITC-FSA in LSECs was significantly reduced in *Glmp*^*gt/gt*^ compared to wild-type mice. Expression of LSEC receptors stabilin-1 (*Stab1*), and mannose receptor (*Mcr1*) was almost similar in liver of *Glmp*^*gt/gt*^ mice and age-matched controls. At the same time, immunostaining revealed differences in the stabilin-1 expression pattern in sinusoids and accumulation of stabilin-1-positive macrophages in *Glmp*^*gt/gt*^ liver. FcγRIIb (*Fcgr2b*), which mediates LSEC endocytosis of soluble immune complexes was widely and significantly downregulated in *Glmp*^*gt/gt*^ liver. Despite increased collagen in space of Disse, LSECs of *Glmp*^*gt/gt*^ mice showed well-preserved fenestrae organized in sieve plates but the frequency of holes >400 nm in diameter was increased, especially in areas with hepatocyte damage. In both genotypes, FITC-FSA also distributed to endothelial cells of spleen and bone marrow sinusoids, suggesting that these locations may function as possible compensatory sites of clearance of blood-borne scavenger receptor ligands in liver fibrosis.

## Introduction

The liver sinusoids are lined with a specialized endothelium, which is vital to the functional homeostasis of the organ [1, 2]. Structurally, the liver sinusoidal endothelial cells (LSECs) are perforated with numerous open, nanosized holes, or fenestrae with a diameter of approximately 50–300 nm [3]. The fenestrae are arranged in sieve plates [4], and cover 2–20% of the cell surface area [5]. LSECs lack an organized basal lamina, and the open fenestrae allow circulating solutes, macromolecules, and colloids direct access to the space of Disse. This function is essential for normal substrate transfer and lipoprotein traffic to and from the hepatocytes. Decreased LSEC porosity is reported in chronic liver disease [6–8] and aging [9], and has been linked to impaired liver uptake of chylomicron remnants and hyperlipoproteinemia [10, 11].

LSECs further have a very high endocytic capacity and are major scavenger cells for spent plasma proteins, oxidized lipoproteins, small, soluble immune complexes, and waste macromolecules from tissue repair and turnover processes [1, 12, 13]. To exert this function, LSECs express a distinct set of endocytosis receptors [14]. The most studied of these receptors are the mannose receptor (CD206), the Fc gamma receptor IIb2 (FcγRIIb2, CD32b), and the two members of the Class H scavenger receptors: stabilin-1, and stabilin-2 [12–15]. FcγRIIb2, stabilin-1, and stabilin-2 are specific for LSECs in healthy liver [15–21], whereas the mannose receptor may to some extent also be expressed in Kupffer cells [12, 20]. However, soluble ligands for the mannose receptor, such as lysosomal enzymes [22, 23], C-terminal procollagen propeptides [24], and collagen alpha chains [25, 26] end up, for a large part in LSECs after intravenous administration. LSECs express several classes of scavenger receptors [14, 20] but have been suggested to rely much on the stabilins for blood clearance of many scavenger receptor ligands [12, 13], including oxidized lipoproteins [27], N-terminal procollagen propeptides [28], hyaluronan (only stabilin-2) [15, 28–30], and formaldehyde-treated serum albumin (FSA) which is a model ligand used to assess scavenger receptor activity in the cells [27, 28, 31, 32]. FcγRIIb2 mediates LSEC uptake of small, soluble immune complexes of the IgG class through clathrin-mediated endocytosis [16, 21]. Most studies on LSEC scavenger functions have been conducted in healthy animals; hence our knowledge about this function in liver disease is largely unknown [12]. However, downregulation of FcγRIIb/CD32b and stabilin-2 is reported in chronic liver disease [12, 33, 34], suggesting that the LSEC endocytic capacity is affected in these conditions.

A range of insults, including toxic damage, viral infection, metabolic, autoimmune, and genetic diseases can lead to chronic liver disease and the development of tissue fibrosis [6, 35]. In the present study, we have used the *Glmp*^*gt/gt*^ mouse [36, 37] to study LSEC scavenger function and morphology in a state of chronic, mild liver injury, inflammation, and collagen accumulation. This mouse model has been thoroughly characterized in a series of studies [36–39] and is suggested as a model for spontaneous, slowly progressing periportal liver fibrosis. All *Glmp*^*gt/gt*^ mice lack the expression of glycosylated lysosomal membrane protein (GLMP) [37], which is a highly conserved, and ubiquitously expressed 404 amino acid protein with no sequence homology to other known proteins [40]. The protein is a single pass N-glycosylated lysosomal membrane protein [41] and an essential subunit of the major facilitator superfamily domain containing 1 (MFSD1) lysosomal membrane protein transporter complex [42]. At birth the *Glmp*^*gt/gt*^ mice appear healthy. However, within a few weeks they develop liver fibrosis and splenomegaly as the dominating phenotype, due to an increased rate of hepatocyte death that starts after birth [37, 39]. Markers of inflammation, apoptosis, fibrosis, and modulation of the extracellular matrix are peaking at approximately 4 months of age with liver injury being more severe in males than in females [39]. Hepatic progenitor cells and hepatic stellate cells are activated prominently around 3 to 4 months of age. At the age of 9 months, the liver shows a balance or mixture of hepatocyte loss and replacement; however, there is an active fibrogenesis throughout life, and after 12 months tumours also start to appear. At 18 months of age approximately 70% of the *Glmp*^*gt/gt*^ mice have developed liver cancer, with similarities to human hepatocellular carcinoma [39]. However, LSEC morphology and functions have not been studied in this animal model.

The aim of our study was therefore to examine how LSEC scavenger function and ultrastructure are affected by the persistent liver damage, inflammation, and fibrosis in the *Glmp*^*gt/gt*^ mice at two different stages in the development of liver disease, i.e. at 4 months when the liver injury and inflammation peaks, and at 9–10 months, shortly before onset of tumour formation. To this end we analysed the in situ sinusoidal ultrastructure of livers of *Glmp*^*gt/gt*^ mice and age-matched *Glmp*^*wt/wt*^ mice, liver expression of four major LSEC endocytosis receptors (stabilin-1, stabilin-2, mannose receptor, FcγRIIb), collagen content in liver, and the in vivo distribution of the scavenger receptor ligand FSA, which is known to be rapidly and predominantly cleared from blood by LSECs in normal liver [22, 31, 32].

## Material and methods

### Animals

*Glmp*^*gt/gt*^ mice (formerly known as *NCU-G1*^*gt/gt*^) [37] and wild-type control mice (*Glmp*^*wt/wt*^) were bred from homozygous parents and housed at the Animal Facility at the Department of Biosciences, University of Oslo (UiO), Norway. The *Glmp*^*wt/wt*^ mice (named WT mice in the following) were descendants of wild-type siblings of *Glmp*^*gt/gt*^ mice, obtained from heterozygous (*Glmp*^*gt/wt*^) breeding. The *Glmp*^*gt/gt*^ mice do not express the *Glmp* gene due to a gene-trap inserted into the first intron of the gene [37]. The mouse model has a C57BL/6 background. The WT and *Glmp*^*gt/gt*^ mice used in the project were accommodated in the same room designed specifically for mice, with a 12h/12h day-night cycle. The animals had free access to water and food (standard chow). In total, 14 WT and 16 *Glmp*^*gt/gt*^ mice were included in the in vivo study. The animals were divided into experimental groups based on genetic status, gender, and age: 1) male WT mice aged 3–6 months (n = 5), 2) male *Glmp*^*gt/gt*^ mice aged 4 months (n = 6), 3) female WT mice aged 4–6 months (n = 5), 4) female *Glmp*^*gt/gt*^ mice aged 4 months (n = 4); 5) male WT mice aged 10 months (n = 4), and 6) male *Glmp*^*gt/gt*^ mice aged 10 months (n = 6). Liver tissue for quantitative real-time PCR (qPCR) and western blots were from 8 *Glmp*^*gt/gt*^ mice and 8 WT mice (all males, aged 4, or 9 months) included in the study of the *Glmp*^*gt/gt*^ phenotype published in [39].

Primary mouse LSECs which were included as controls in western blot analyses were from male C57Bl/6JRj mice (Janvier Lab, France). Cells were isolated by liver perfusion with collagenase buffer according to the protocol in [43]. The whole experimental procedure was performed post-mortem in mice euthanized by cervical dislocation. The experimental protocol and animal handling for ex vivo liver perfusion and cell isolation were approved by the competent institutional authority at the UiT-The Arctic University of Norway, licensed by the National Animal Research Authority at the Norwegian Food Safety Authority (Approval ID: 09/22).

### In vivo endocytosis experiments and sampling of tissue

Formaldehyde-treated bovine serum albumin (FSA) was prepared as described in [44] and labelled with fluorescein isothiocyanate (FITC) [45]. FITC-labelled FSA (FITC-FSA), 2 μg/g body weight, in 0.9% sterile NaCl, was injected into the tail vein of anesthetized *Glmp*^*gt/gt*^ mice, and age and gender-matched WT mice. The animals were anaesthetized with an intraperitoneal injection of a combination of zolazepam hydrochloride, tiletamine hydrochloride (Zoletil forte vet^®^, Virbac), xylazine (Narcoxyl vet^®^, Intervet), and fentanyl (Fentadon vet^®^, Eurovet) in a cocktail designed for mouse [46]. One ml of this anaesthetic contains zolazepam hydrochloride (3.3 mg), tiletamine hydrochloride (3.3 mg), xylazine (0.45 mg), and fentanyl (2.6 μg) in 0.9% NaCl. Ten min after injection of ligand, the mice were euthanized by CO_2_ inhalation while still anaesthetized. Immediately following termination, the dead animal was perfusion fixed via the left heart ventricle with outlet via the right atrium; first 10 ml of phosphate buffered saline (PBS pH 7.4) was injected, immediately followed by 10–20 ml of 4% formaldehyde in PBS (pH 7.4) and organs harvested. Samples for fluorescence microscopy, histochemistry and immune histochemistry (IHC) were prepared for paraffin embedding. As the *Glmp*^*gt/gt*^ mice show the same pathology throughout the liver [37, 39] the big left liver lobe was used in all experiments.

For electron microscopy, samples from the perfusion-fixed left liver lobe were cut in pieces of approximately 1mm^3^ and further fixed in 2.5% glutaraldehyde and 4% formaldehyde in PHEM buffer (w/v: 1.81% PIPES, 0.65% HEPES, 0.38% EGTA, 0.1% MgSO_4_), pH 7.

### Fluorescence microscopy and quantitative image analysis of FITC-FSA uptake in the liver

Paraffin-embedded samples from the left liver lobe of all mice included in the in vivo study (n = 30) were sectioned at 4 μm thickness, de-paraffinized, rehydrated and incubated for 5 min with 4′6-diamidino‐2-phenylindole (DAPI; Sigma-Aldrich, Cat. No D8417) diluted 1:50.000 in PBS to stain the cell nuclei. The sections were mounted in Vectashield mounting medium for fluorescence (Vector Laboratories, Cat. No H-1000). Approximately 25 to 30 images were captured per section using a Zeiss Axio Zoom V16 Stereo Zoom Microscope (Carl Zeiss, Oberkochen, Germany). The imaging was done by first creating a map/overview of the entire liver section in the DAPI channel. Positions were then registered randomly on the map to automatically allow the microscope to image the area of the selected positions at 260x magnification. The images taken covered approximately 50% to 70% of the liver section, excluding areas with tissue loss or folds. The imaging process secured an unbiased sampling process by selecting positions on the map without seeing the specific FITC fluorescence.

To clearly distinguish specific fluorescence resulting from FITC-FSA uptake in cells from autofluorescence in the liver tissue, the images were deconvolved using the deconvolution software Huygens Essential (version 18.10) part of the Huygens Software SVI Huygens Pro (Scientific Volume Imaging). Quantitative image analysis to calculate the percentage of tissue area covered by FITC-fluorescence was performed with Fiji (Image J, version 2.0.0, Java 1.8.0). We here used image segmentation thresholding to measure the area in pixels. The original (non-deconvolved) images were used to measure the total area of the liver tissue excluding the luminal area of large vessels and sinusoids. In contrast, the deconvolved images were used to measure the FITC-positive area. The percentage of liver tissue area covered by FITC-fluorescence was calculated by dividing the FITC-positive area calculated from the deconvolved image by the total liver area calculated from the original image. This process was automated using command lines in macros in Fiji.

### Fluorescence microscopy of FITC-FSA uptake in spleen and bone marrow

Paraffin sections from spleen and bone marrow of WT and *Glmp*^*gt/gt*^ mice were de-paraffinized, rehydrated, incubated for 5 min with DAPI, mounted in Vectashield, and imaged in a Zeiss Axio Zoom V16 Stereo Zoom Microscope. Overview maps of the sections of spleen were made as described for liver. Then 20 images per section were taken at 125x at randomly pre-set locations for image analysis as described under “Fluorescence microscopy and quantitative image analysis of FITC-FSA uptake in the liver”.

### Histochemistry

Paraffin sections from all livers (n = 30) were stained with haematoxylin and eosin for histopathological assessment, and with Picro Sirius Red (PSR) staining kit (Abcam, Cat. No ab150881) for examination of collagen accumulation. Approximately 18 images per section were captured at 125x magnification using the Zeiss Axio Zoom V16 Stereo Zoom microscope to measure the area covered by collagen in liver sections. The PSR stained area over the total liver tissue area was measured using thresholding in Fiji as described in the section “Fluorescence microscopy and quantitative image analysis of FITC-FSA uptake in the liver”.

### Correlative imaging of FITC-fluorescence and collagen in paraffin sections

Two sets of paraffin sections, sectioned in parallel, were prepared from all livers (n = 30). One set of sections was prepared for fluorescence microscopy and the other set was stained with a PSR staining kit to visualize collagen. Images were taken with the Zeiss Axio Zoom V16 Stereo Zoom microscope using the multi-image mode (splitter mode) on ZEN 2012 SP2 (blue edition) to match and image FITC-fluorescence and PSR staining within the exact location in the sections.

### Immunohistochemistry

#### Immune fluorescence

Liver paraffin sections from the male mice included in the in vivo study (4–6 mice per age group and genotype) were placed on Superfrost Plus Adhesion Microscope Slides (Thermo Fisher Scientific), incubated overnight at 60°C, de-paraffinized in xylene, and rehydrated through a graded ethanol series. Antigen retrieval was done by microwaving 4x 5 min in 0.01 M citrate buffer (pH = 6); time was extended to 4x 10 min for stabilin-1 staining. Sections were blocked in 3% BSA (AppliChem, Cat. No A1391), or 10% Donkey serum (Sigma, Cat. No D9663) in PHEM buffer, pH 7, for 1 h at room temperature, and incubated overnight at 4°C with goat anti-mouse FcγRII/RIII (CD32/CD16, R&D Systems, Cat. No AF1460; working concentration: 4 μg/ml), goat anti-human MMR/CD206 (R&D Systems, Cat. No AF2534; 5 μg/ml), rabbit anti-human stabilin-1 (Atlas, Cat. No HPA005434; 3 μg/ml), goat anti-mouse VSig4 (Bio-Techne, Cat. No AF4674; 5 μg/ml), rabbit anti-mouse CD68 (Abcam, Cat. No ab125212; 2 μg/ml), and non-immune goat IgG (R&D Systems, Cat. No AB-108.C), or rabbit IgG (Dako, Cat. No X0936) at similar concentration as primary antibody. Two antibodies against stabilin-2 were also tested but did not function on the mouse paraffin liver sections in our study (rat anti-mouse stabilin-2, MBL, Cat. No D317-3, and rabbit anti-human stabilin-2, Atlas, Cat. No HPA026871).

The specificity tests of the goat anti-human MMR/CD206 (R&D Systems, Cat. No AF2534), and the rabbit anti-human stabilin-1 (Atlas, Cat. No HPA005434) antibodies for the respective mouse receptors are presented in Supporting Information (S1 and S2 Figs).

Secondary antibodies used were Alexa Fluor (AF)-555 Donkey anti-goat (Abcam, Cat. No 21432; 4 μg/ml), DyLight 550 Goat anti-rabbit (Invitrogen, Cat. No SA5-10033, 3 μg/ml), AF555 Goat anti-rat (Invitrogen, Cat. No A21434; 4 μg/ml), AF555 Donkey anti-rabbit (Invitrogen, Cat. No A31572; 4 μg/ml), and AF647 Donkey anti-rabbit (Invitrogen, Cat. No A31573; 4 μg/ml). Antibodies were diluted in blocking buffer. Cell nuclei were stained with DAPI, and sections were mounted in Vectashield and imaged with a Zeiss LSM 800 confocal laser scanning microscope equipped with 25x (NA 0.55), and 40x (NA 1.2) water objectives. The percent positively stained area over the total liver tissue area on 3–10 overview images (25x objective) per section was measured using thresholding in Fiji.

#### ICAM-1 staining

Liver paraffin sections from 12 male mice (3 animals per age group and genotype) were labelled. Antigen retrieval was done by microwaving the sections 4x 5 min in 0.01 M citrate buffer (pH = 6) with 0.05% Tween. All antibodies were diluted in 2% BSA in PBS. Endogenous peroxidase was quenched in 3% H_2_O_2_ in methanol, and endogenous biotin with Biotin-Blocking System (Agilent, Cat. No X0590). Sections were incubated overnight at 4°C with Armenian hamster anti-mouse CD54 (ICAM-1) (5 μg/ml; Invitrogen—Thermo Fisher Scientific, Cat. No MA5405) or Armenian hamster IgG isotype control (5 μg/ml; Invitrogen—Thermo Fisher Scientific, Cat. No 14-4888-81). After rinsing in PBS with 0.05% Tween, sections were incubated for 30 min at RT with biotin-streptavidin-conjugated goat anti-Armenian hamster IgG (H+L, eBioscience, Cat. No 13–411385; 1.25 μg/ml), washed and incubated for 30 min with Streptavidin peroxidase (Agilent, Cat. No P0397). A diaminobenzidine substrate chromogen system kit (BD Pharmingen, Cat. No 550880) was used to visualize a positive reaction. Sections were counter-stained with haematoxylin, and images taken in a Nikon Eclipse TE2000-U Inverted Microscope.

### Scanning electron microscopy (EM)

Liver samples from 12 mice, including 4 WT male mice, aged 4–6 months, 4 *Glmp*^*gt/gt*^ male mice, aged 4 months, and 4 *Glmp*^*gt/gt*^ male mice, aged 9–10 months were prepared for scanning EM. The samples were from the mice included in the FITC-FSA distribution study which showed FITC-FSA uptake in the liver closest to the median value for each group. Perfusion fixed (4% formaldehyde in PBS) tissue samples from the left big liver lobe were cut in approximately 1mm^3^ tissue blocks, and further fixed in 4% formaldehyde and 2.5% glutaraldehyde in PHEM buffer, pH 7, as described under “In vivo endocytosis experiments and sampling of tissue”. The aldehyde fixed tissue blocks were post-fixed in 1% osmium tetroxide in water (Electron Microscopy Sciences), dehydrated through a graded series of ethanol (30–100%), transferred to liquid nitrogen and gently cracked open to expose the inner lining of the sinusoids, then transferred back to 100% ethanol, and chemically dried using hexamethyldizilasane (HMDS; Sigma-Aldrich) [47]. The dried tissue blocks were mounted on aluminium stubs, sputter coated with gold/palladium alloy (Polaron SC 7640, Quorum Technologies Ltd, Laughton East Sussex, UK) and examined in a Zeiss Sigma scanning electron microscope (Carl Zeiss), at 2kV. From each liver, images were captured at low (<1000x), medium (2000–10,000x) and high magnification (20,000x) from at least three blocks per liver, picked at random, for overview studies of the liver architecture and detailed analyses of the sinusoid and LSEC morphology. Images taken at 20,000x magnification (11–29 images per liver) were used for quantitative measurements of LSEC fenestrae in situ. The images were randomly taken from the sinusoids that had been cracked open to expose the sinusoidal luminal LSEC surface. Fenestrae (open holes < 400 nm in diameter [3]) and gaps (open holes > 400 nm in diameter) were manually counted on 206 SEM images at 20,000x magnification using Digimizer image analysis software, and the number of fenestrae or gaps per μm^2^ were calculated. Open holes < 30 nm in diameter were excluded from the analysis.

### Real-time quantitative PCR (qPCR)

Liver samples from 4, or 9 months old *Glmp*^*gt/gt*^ and age-matched WT male mice were snap-frozen in liquid nitrogen [39], stored at -70°C until processing, and homogenized using Magnalyser green beads (Roche, Cat. No 3358941001) with the Precellys 24 Tissue homogenizer (Bertin Technologies) for 23 sec at 6500 rpm in 800 μl of homogenization solution (from Maxwell 16 miRNA tissue kit, Promega, Cat. No AS1470). RNA was extracted with Maxwell 16 miRNA tissue kit, including DNase I treatment, in a Maxwell 16 Instrument (AS1000) reconfigured with the Maxwell 16 High Strength LEV Magnetic Rod and Plunger Bar Adaptor. RNA purity and concentration were assessed with Nanodrop: A260/280 values were 1.91–1.97 and RNA concentrations were 91–600 ng/μl. The integrity of total RNA was determined with the Agilent 2100 Bioanalyzer system; RNA integrity numbers (RIN) were above 7 except for one sample with RIN 6.7. The cDNA libraries were prepared with the QuantiTect Reverse Transcription Kit (Qiagen, Cat. No 205311) using 800 ng RNA per reaction. Information about target, primers, and experimental validation is shown in S1 and S2 Tables. Reference Gene Panel Mouse SYBR (TATAA Biocenter AB, Cat. No A102) was used to choose the most appropriate reference genes to compare fibrotic and normal liver tissue across the two age groups. Hypoxanthine-guanine phosphoribosyl transferase (HPRT) showed the most consistent expression across the groups and was chosen to normalize target gene expression. PCR reactions were performed in duplicates using FastStart SYBR Green Master (Sigma Aldrich, Cat. No 4673484001) in LightCycler 96 (Roche Life Science).

### Western blot

HEK293 cells were obtained from ATCC. HEK293 stably expressing mouse stabilin-1 were kindly provided by Dr. Staffan Johansson (University of Uppsala, Sweden); mouse stabilin-1 cDNA was cloned in the pEF6V5His-TOPO vector (Merck) via Spe1-Not1, and cells transfected by lipofection. Control cells were transfected locally by lipofectamine using the empty vector.

HEK293 cells, primary mouse LSECs (CD146-MACS purified; [43]), and snap frozen mouse liver tissue [39] was solubilized in RIPA buffer (Thermo Scientific, Cat. No 89900) containing protease inhibitor cocktail (Roche, Cat. No 04693159001), vanadate, pepstatin A, and N-ethylmaleimide. Protein concentration was measured by Direct Detect Spectrometer (Millipore), and samples sonicated, reduced, and heated at 70°C for 10 min. Samples for analysis of FcγRII were run on SDS-PAGE using Nupage 4–12% Bis-Tris gels (Invitrogen), while samples for stabilin-1 and mannose receptor analyses were run on 3–8% NuPage Tris-Acetate gels (Invitrogen) according to the manufacturer´s protocol, together with the recommended protein standards (HiMark Pre-stained protein standard, Cat. No LC5699; Himark Unstained protein standard together with Coomassie blue staining, Cat. No LC5688; Precision Plus protein Dual color standards, Cat. No 1610374, Bio-Rad; MagicMark western protein standard, Cat. No LC5603, Invitrogen). Blotting was done onto 0.45 μm PVDF transfer membranes (Thermo Scientific, Cat. No 88518), and unspecific signal was blocked by incubation with 1xTBS with 0.1% Tween 20 (TBST) and 5% low-fat powder milk (blocking buffer), for 1h at RT, followed by incubation with primary antibody overnight at 4°C in blocking buffer. The washed blots were incubated with HRP-conjugated secondary antibody in blocking buffer, for 1 h, at RT. The stained proteins were visualized with SuperSignal West Pico Plus chemiluminescent substrate (Thermo Scientific, Cat. No 34580) and imaged in ImageQuant LAS 4000.

Primary antibodies were goat anti-human MMR/CD206 (R&D; 1 μg/ml), rabbit anti-human stabilin-1 (Atlas; 1 μg/ml), goat anti-mouse FcγRII/RIII (CD32/CD16, R&D; 0.1 μg/ml), and rabbit polyclonal beta-actin antibody (Abcam, Cat. No ab8227; 0.2 μg/ml; loading control). Secondary antibodies were donkey anti-goat IgG (H+L) cross-absorbed, HRP (Invitrogen, Cat. No A16005, diluted 1:10.000), and goat anti-rabbit (IgG), HRP (Abcam, Cat. No ab205718, diluted 1:40.000). In the analyses of mannose receptor and stabilin-1, which are high molecular weight receptors, the blots were cut at approximately 60 kDa and the beta-actin loading control performed on the bottom part. In the analyses of FcγRII/RIII, beta-actin staining was performed on the stripped blot (stripping buffer: 0.15% glycine, 0.1% SDS, 1% Tween 20, pH 2.2).

### Statistical analysis and software

Statistical analyses were done in Microsoft Excel (Microsoft) and GraphPad Prism 8 (GraphPad Software). Mann Whitney U test was used to compare two datasets, and One-way non-parametric ANOVA on ranks (Kruskal–Wallis test) was used for comparison of several datasets. The qPCR data were analysed with Genex 7.0 software (MultiD Analyses AB). NormFinder [48] and GeNorm [49] software were used to find the most appropriate reference genes. P-values < 0.05 were considered statistically significant. Image panels were made in Adobe Illustrator CC (Adobe Systems Inc.) and Affinity Publisher (Serif Europe Ltd) and graphs in GraphPad Prism 8.

## Results

### Pathology assessment of liver samples

**Fig 1:**
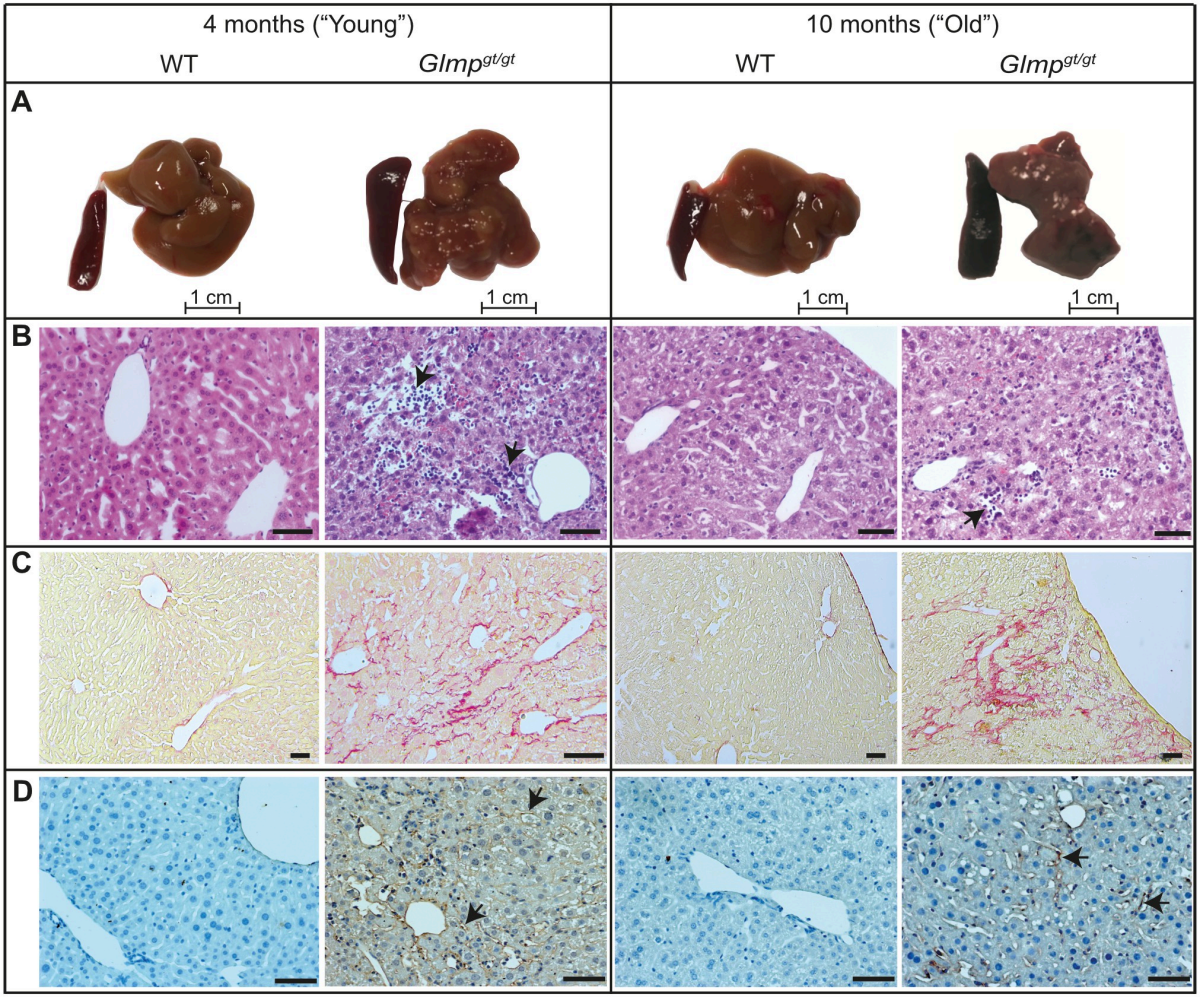
Gross morphology and histological appearance of *Glmp*^*gt/gt*^ mouse liver. A) Representative images of liver and spleen from 4 and 10 months old male *Glmp*^*gt/gt*^ and WT mice. The *Glmp*^*gt/gt*^ mice had a tuberous liver appearance and splenomegaly, consistent with previous reports [37, 39]. B-D) Paraffin-embedded liver sections stained with B) haematoxylin and eosin (arrows point to tissue injury and infiltration of inflammatory cells in the *Glmp*^*gt/gt*^ liver sections), C) Picro Sirius Red collagen stain, and D) an antibody to ICAM-1. Positive ICAM-1 staining is seen as brown pigment in sinusoids (arrows) and veins in the *Glmp*^*gt/gt*^ liver sections. Scale bars in B-D: 50 μM.

In agreement with former reports [36, 37, 39], the *Glmp*^*gt/gt*^ mice showed a nodulated liver, and splenomegaly (Fig 1A). Histological assessment of HE and PSR stained liver sections revealed multifocal hepatocyte necrosis in the *Glmp*^*gt/gt*^ livers, with infiltration of polymorph-nucleated and mononuclear inflammatory cells in the injured areas and development of liver fibrosis (Fig 1B and 1C). The most severe pathology was observed in the liver of 4 months old *Glmp*^*gt/gt*^ male mice, as also previously reported in [39]. Liver inflammation and fibrosis were most pronounced in the periportal region, extending to the central vein and liver capsule in some areas. WT mice showed normal liver histology, with scarce amount of collagen in the portal tract, central vein, and liver capsule.

ICAM-1 is expressed only at a low level in the sinusoids of healthy mouse liver, but can be upregulated in inflammation [50]. *Glmp*^*gt/gt*^ mice showed enhanced ICAM-1 staining in the liver sinusoids compared to age-matched controls (Fig 1D).

### Analysis of FITC-FSA uptake and collagen content in tissue sections from *Glmp*^*gt/gt*^ and WT liver

**Fig 2:**
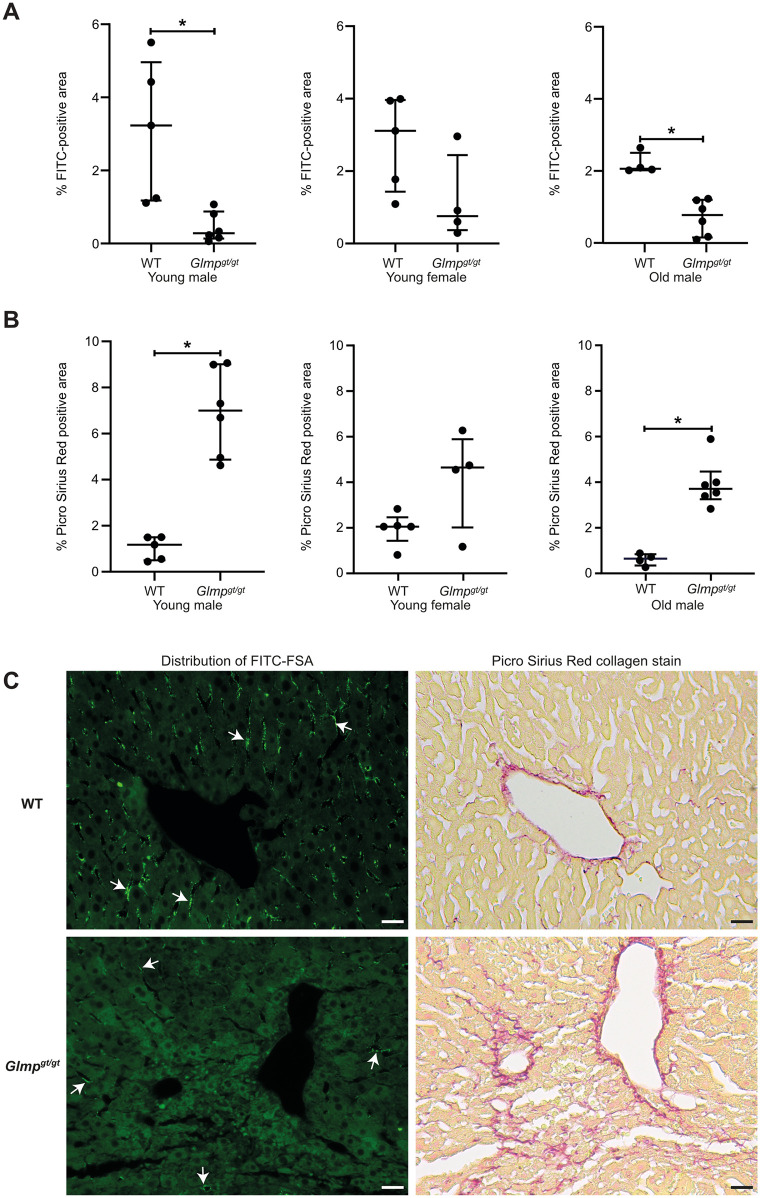
FITC-FSA uptake and collagen content in the liver from *Glmp*^*gt/gt*^ versus WT mice. A) Uptake of FITC-FSA in liver of *Glmp*^*gt/gt*^ mice (n = 16) and WT mice (n = 14), presented as % FITC-positive area in liver sections. Organs were harvested 10 min after intravenous injection of FITC-FSA (dose: 2 μg/g body weight). Age of young groups: *Glmp*^*gt/gt*^, 4 months; WT, 3–6 months. Old groups (both genotypes): 9–10 months. Each dot represents one animal, median values are presented as horizontal lines, and upper and lower lines represent the interquartile range. Mann Whitney U test compared results from *Glmp*^*gt/gt*^ and age- and gender-matched WT mice. Young males: *p-value < 0.01; young females: not statistically significant; old males: *p-value < 0.01. B) Collagen content (% Picro Sirius Red stained area) in the liver of *Glmp*^*gt/gt*^ mice (n = 16) and WT mice (n = 14), quantified by image analysis as described in Methods. Each dot represents one animal, median values are presented as horizontal lines, and upper and lower lines represent interquartile range. Mann Whitney U test: young males, *p-value < 0.01; young females, not statistically significant; old males, *p-value < 0.01. C) Distribution of FITC-FSA (bright green fluorescence, white arrows), and collagen (red stain) in parallel sections of liver samples from 4 months old WT and *Glmp*^*gt/gt*^ mice. In the WT liver, FITC-fluorescence is seen as small bright green dots in the endothelial cells of most sinusoids. At the same time the parallel Picro Sirius Red stained section shows only a low amount of collagen in the sinusoids. In the *Glmp*^*gt/gt*^ mouse liver uptake of FITC-FSA was low or absent in areas with collagen accumulation. Scale bars: 20 μm.

To examine LSEC scavenging function in vivo, FITC-FSA was injected into the tail vein of 4 and 9–10 months old *Glmp*^*gt/gt*^ mice and age-matched WT controls. The animals were euthanized 10 min post injection for organ analysis. FSA is a soluble scavenger receptor ligand known to be rapidly cleared from blood after intravenous injection in rats and mice. It distributes mainly to liver, where it is primarily endocytosed by LSECs [22, 31, 32]. For this reason FITC-FSA has been used as a functional LSEC marker [1]. To estimate the relative uptake of FITC-FSA in the liver of *Glmp*^*gt/gt*^ mice versus WT mice, we measured the area covered by FITC-fluorescence in liver sections. The ligand was observed to distribute along the hepatic sinusoids in a pattern typical for uptake in LSECs [31], different from the staining pattern of the macrophage markers CD68 and VSIG4 (S3 and S4 Figs (Z-stack video)). However, a minor uptake in sinusoidal macrophages cannot be excluded (S3B Fig) as these cells are superimposed in the sinusoidal wall. Uptake of FITC-FSA was significantly lower in male *Glmp*^*gt/gt*^ livers compared to age-matched WT males in both age groups (Fig 2A). The median value (% liver tissue area covered by FITC-fluorescence) was also lower in the female *Glmp*^*gt/gt*^ mice compared to the female WT group. Still, the difference was not statistically significant due to high variation in the *Glmp*^*gt/gt*^ group (Fig 2A).

Collagen content in the liver was measured as % PSR positive area in PSR-stained liver sections, and was higher in all *Glmp*^*gt/gt*^ livers, compared to WT livers, except for one sample in the *Glmp*^*gt/gt*^ female group (same animal that showed normal uptake of FITC-FSA) **(**Fig 2B). The median value was highest in the 4 months old male *Glmp*^*gt/gt*^ group (7%, compared to 1.3% in the age- and gender-matched WT group, p < 0.01).

To investigate the relationship between FITC-FSA uptake and collagen content in liver, we examined parallel liver sections from *Glmp*^*gt/gt*^ (n = 16) and WT (n = 14) mice, where one section was prepared for fluorescence microscopy and one for PSR staining from each liver. In WT livers, which all showed only scarce amounts of collagen staining, uptake of FITC-FSA was observed in nearly all sinusoids. In contrast, in the *Glmp*^*gt/gt*^ livers, uptake of FITC-FSA was low or absent in lobular regions with collagen accumulation (Fig 2C), while present in other areas with less collagen staining.

### Expression of LSEC endocytosis receptors in *Glmp*^*gt/gt*^ and WT mice

**Fig 3:**
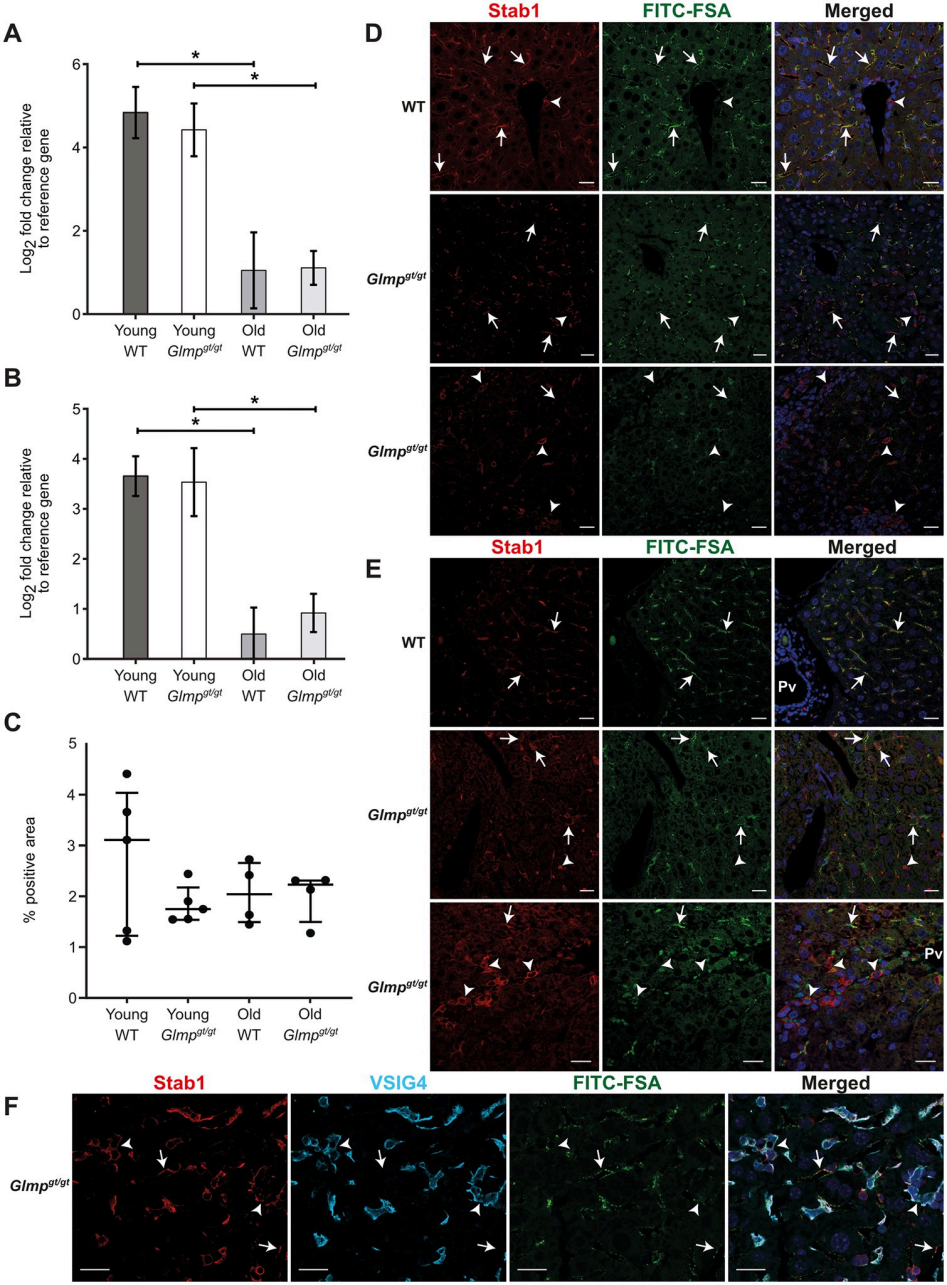
Stabilin-1 and stabilin-2 expression in liver from *Glmp*^*gt/gt*^ and WT mice. A-B) Quantitative PCR analysis of *Stab1* (A) and *Stab2* (B) expression in liver tissue from 4 months (“young”) and 9 months (“old”) WT and *Glmp*^*gt/gt*^ male mice (young: n = 4 per group; old: n = 3 per group). Error bars represent standard deviation. Results were not significantly different with Mann Whitney U test or the Kruskal-Wallis test. C) Quantitative image analysis of stabilin-1-stained liver sections from WT and *Glmp*^*gt/gt*^ mice, presented as % positively stained tissue area. Groups: Young WT, 3–6 months (n = 5); young *Glmp*^*gt/gt*^, 4 months (n = 5); old WT, 9–10 months (n = 4); and old *Glmp*^*gt/gt*^, 9–10 months (n = 4). Each dot represents one animal, the median value for each group is presented as a horizontal line, and the upper and lower lines represent the interquartile range. Statistical analysis showed no significant differences between groups (One-way non-parametric ANOVA on ranks/Kruskal-Wallis test). D-E) Distribution pattern of stabilin-1 (red fluorescence) in liver of D) 4 months old, and E) 9–10 months old *Glmp*^*gt/gt*^ and WT mice injected intravenously with FITC-FSA (2 μg/g body weight, 10 min monitoring time). Scale bars: 20 μμm. In the WT mice (both age groups) stabilin-1 was widely distributed in the sinusoids, highly colocalizing with FITC-FSA (arrows in D-E). At the same time, a few stabilin-1 positive, FITC-negative cells were observed in the portal tract (arrowhead in D, WT row). In the *Glmp*^*gt/gt*^ mice, stabilin-1 staining was seen in FITC-positive cells in the liver sinusoids (arrows in D-E) and FITC-negative inflammatory cell aggregates (arrowheads in D-E). F) Co-localisation of stabilin-1 (red fluorescence) with the macrophage marker VSIG4 [51] (light blue fluorescence, arrowheads) in *Glmp*^*gt/gt*^ liver. Arrows point to positive stabilin-1 staining of sinusoidal endothelial cells, which are VSIG4 negative [20]. Scale bar: 20 μm.

LSECs express several scavenger receptors [14, 20]. Of these, stabilin-2 is reported to mediate endocytosis of FSA [27, 28] and the ligand also binds to stabilin-1 [27]. Quantitative PCR analysis for *Stab1* (Fig 3A) and *Stab2* (Fig 3B) showed similar expression of the two receptors in age-matched *Glmp*^*gt/gt*^ and WT livers, and immune labelling of liver sections for stabilin-1 followed by quantitative image analysis did not reveal a significant difference between groups in % positively stained tissue area (Fig 3C). This was supported by western blot analyses of stabilin-1 expression in liver lysates from both genotypes which did not show marked differences in protein expression between WT and *Glmp*^*gt/gt*^ mice (S2B and S2C Fig). However, the staining pattern was different in the *Glmp*^*gt/gt*^ livers in areas with inflammation compared to the more normal part of the liver. In both genotypes, stabilin-1 was found to colocalize with FITC-FSA in sinusoidal lining cells, indicating LSEC expression of the receptor (arrows in Fig 3D–3F). Close to the portal vein stabilin-1 was also observed in scattered FITC-negative single cells (arrowhead in Fig 3D-WT row). In the *Glmp*^*gt/gt*^ liver samples, intense stabilin-1 staining was further observed in aggregates of (mostly) FITC-negative mononuclear inflammatory cells (arrowheads in Fig 3D and 3E, *Glmp*^*gt/gt*^ rows). These cells were identified as macrophages using the marker VSIG4 [51] (Fig 3F). Unfortunately, immune staining for stabilin-2 did not function in our study.

**Fig 4:**
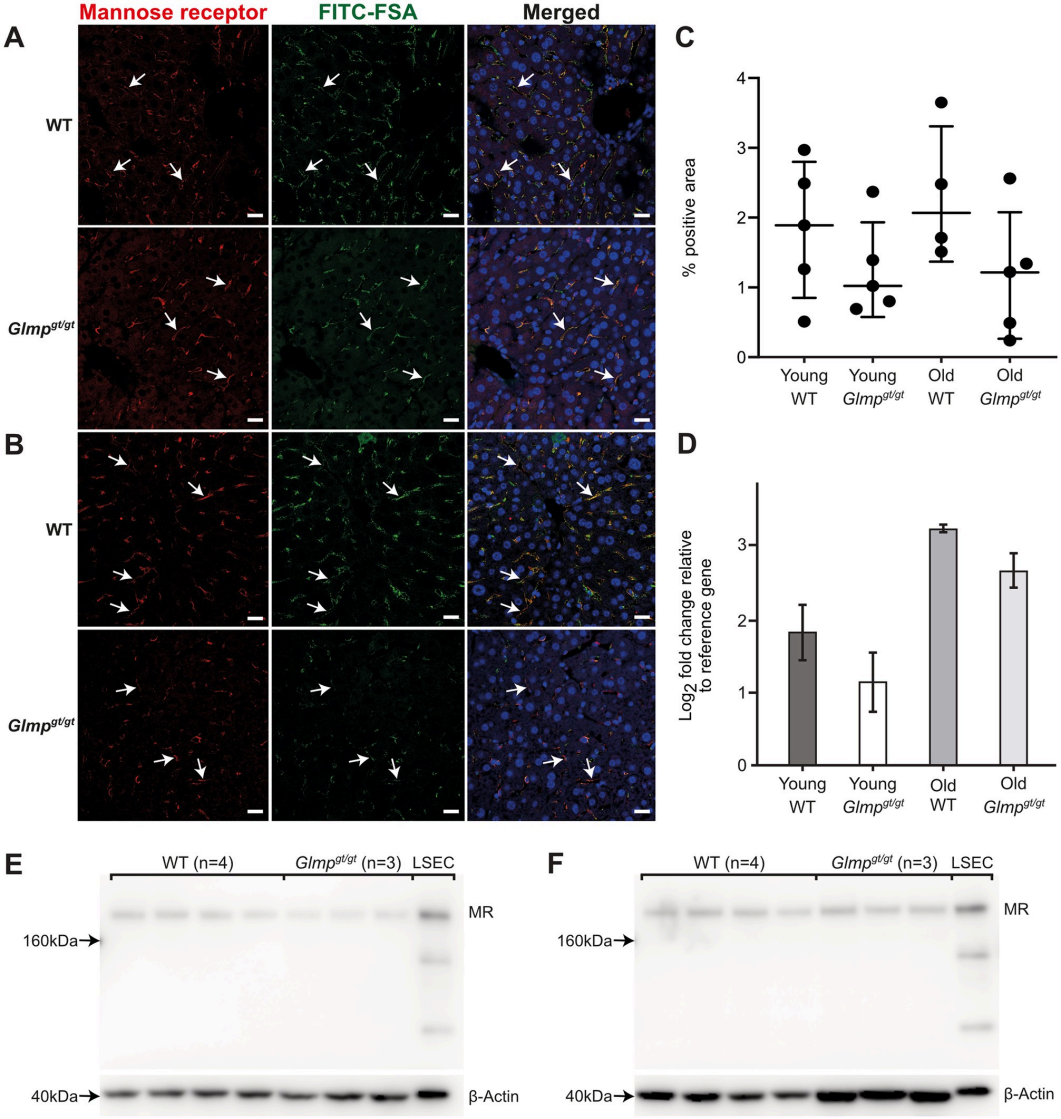
Mannose receptor expression in liver from *Glmp*^*gt/gt*^ and WT mice. A-B) Mannose receptor expression in liver sinusoids of A) 4 months old, and B) 9 months old WT and *Glmp*^*gt/gt*^ male mice. Positive mannose receptor staining (red fluorescence, arrows) was seen in the sinusoids, in the same cells that had endocytosed FITC-FSA (green fluorescence). Nuclei are stained with DAPI (blue). Scale bars: 20 μm. C) Quantitative image analysis of mannose receptor staining (% positive area) in liver sections from WT and *Glmp*^*gt/gt*^ mice. Groups: Young WT, 3–6 months (n = 5); young *Glmp*^*gt/gt*^, 4 months (n = 5); old WT, 9–10 months (n = 4); and old *Glmp*^*gt/gt*^, 9–10 months (n = 5). Each dot represents one animal, the median value for each group is presented as a horizontal line, and upper and lower lines represent the interquartile range. One-way non-parametric ANOVA on ranks (Kruskal-Wallis test) showed no significant differences between groups. D) *Mcr1* mRNA expression (qPCR) in liver tissue from 4 months (“young”) and 9 months (“old”) WT and *Glmp*^*gt/gt*^ male mice (young: n = 4 per group; old: n = 3 per group). Error bars represent standard deviation. Results were not significant in non-parametric tests. E-F) Western blots showing mannose receptor expression in whole liver lysates from E) 4 WT mice and 3 *Glmp*^*gt/gt*^ mice, aged 4 months, and F) 4 WT mice and 3 *Glmp*^*gt/gt*^ mice, aged 9 months, all male. LSEC: Mouse liver sinusoidal endothelial cell lysates C57Bl/6JRj, WT). Protein loaded per lane: Liver lysates, 25 μg; LSEC, 5 μg. Beta-actin loading control was performed on the bottom part of the blots.

We further examined the expression of two other major endocytosis receptors in LSECs, the mannose receptor (*Mrc1*) [22, 25, 52, 53], and the FcγRIIb2 (*Fcgr2b*) [16, 21] by immunohistochemistry, qPCR, and western blot. The distribution of the mannose receptor in the liver from 4 months old, and 9–10 months old *Glmp*^*gt/gt*^ and WT mice is shown in Fig 4. Positive staining was observed along the wall of the liver sinusoids in all groups, co-localizing with FITC-FSA (Fig 4A and 4B). Quantitative image analysis of mannose receptor staining (Fig 4C), supplemented with qPCR and western blot analyses of *Mrc1* gene (Fig 4D) and protein (Fig 4E and 4F) expression in liver tissue did not reveal significant differences between *Glmp*^*gt/gt*^ and age-matched WT mice. The mannose receptor band appeared around 180–200 kDa (Fig 4E and 4F, S1 Fig), corresponding to the reported size of 180 kDa in rat and pig LSEC [25].

**Fig 5:**
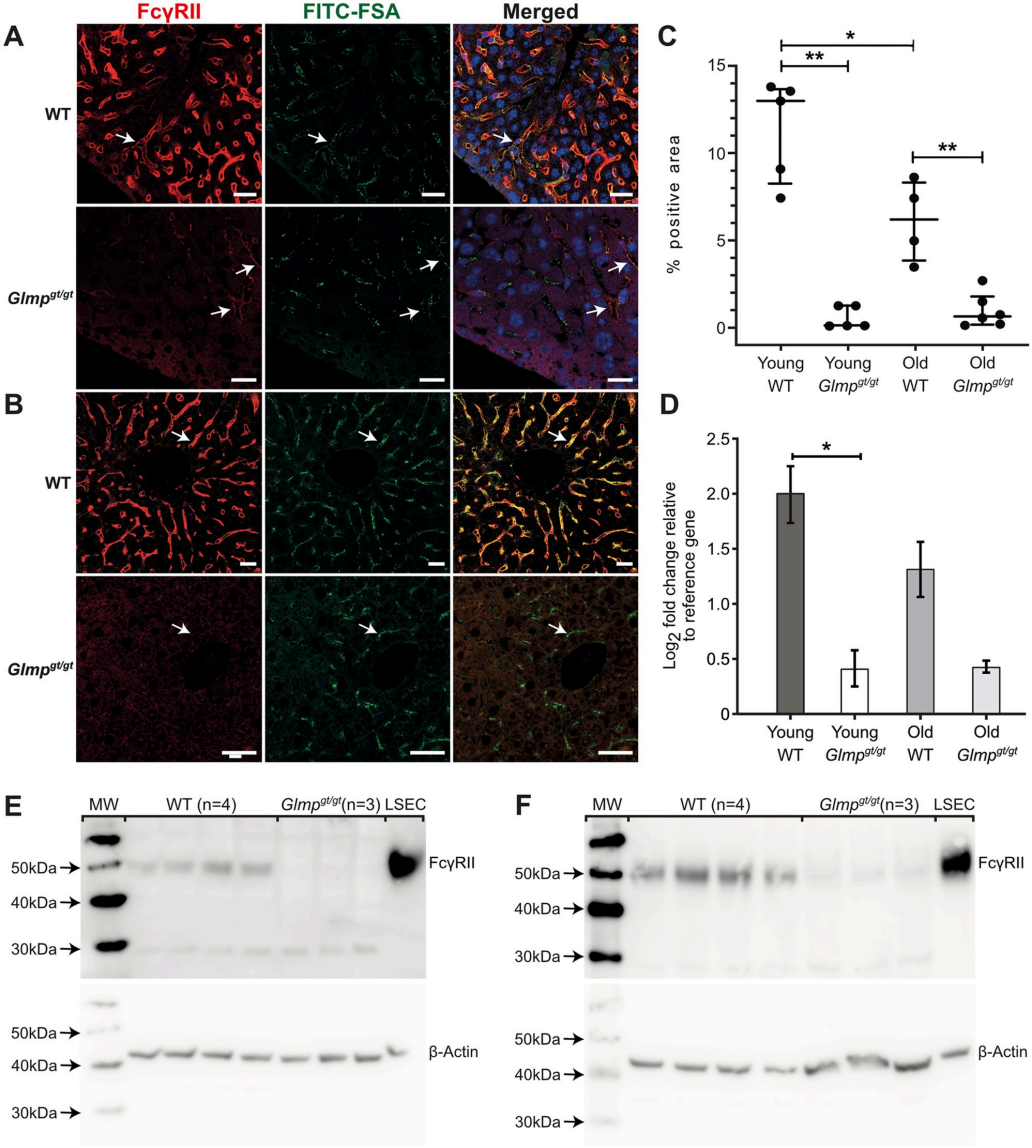
FcγRIIb expression in liver from *Glmp*^*gt/gt*^ and WT mice. A-B) FcγRII expression in liver sinusoids of A) 4 months old, and B) 9–10 months old *Glmp*^*gt/gt*^ and WT male mice. In WT livers, positive immunostaining (red fluorescence) was seen along the sinusoids in the same cells that had taken up FITC-FSA (green fluorescence), while the expression was low or absent in *Glmp*^*gt/gt*^ livers, including cells that had taken up FITC-FSA (arrows). Nuclei in A) are stained with DAPI (blue). Scale bars: 20 μm. C) Quantitative image analysis of FcγRII staining (% positive area) in liver sections from WT and *Glmp*^*gt/gt*^ mice. Groups: Young WT, 3–6 months (n = 5); young *Glmp*^*gt/gt*^, 4 months (n = 5); old WT, 9–10 months (n = 4), and old *Glmp*^*gt/gt*^, 9–10 months (n = 6). Each dot represents one animal. Medians are presented as horizontal lines, and upper and lower lines represent interquartile range. *p-value < 0.05, **p-value < 0.01, One-way non-parametric ANOVA on ranks (Kruskal-Wallis test). D) *Fcgr2b* expression (qPCR) in liver tissue from 4 months (“young”) and 9 months (“old”) WT and *Glmp*^*gt/gt*^ male mice (young: n = 4 per group; old: n = 3 per group). *p-value < 0.05 (Mann Whitney U test). Error bars represent standard deviation. E, F) Western blots showing FcγRII expression in whole liver lysates from E) 4 WT mice and 3 *Glmp*^*gt/gt*^ mice, aged 4 months, and F) 4 WT mice and 3 *Glmp*^*gt/gt*^ mice, aged 9 months, all male. LSEC: Mouse liver sinusoidal endothelial cell lysates (C57Bl/6JRj, WT). Protein loaded per lane: Liver lysates, 25 μg; LSEC, 5 μg. Beta-actin loading control was performed on the stripped blots.

FcγRIIb2, which is a splice variant of FcγRIIb is the only Fc receptor expressed on LSECs [16], and LSECs represent the main reservoir of this receptor in liver [21]. Staining of liver sections with a rabbit polyclonal antibody to mouse FcγRII/RIII (CD32/CD16) showed widespread, strong, specific fluorescence along the sinusoids in all WT livers, and the distribution followed the same pattern as the FITC-FSA uptake, indicating LSEC staining, while labelling of *Glmp*^*gt/gt*^ liver showed a marked, widespread, and significant downregulation of receptor expression in all samples (Fig 5A–5C). The receptor was also absent in sinusoids that had accumulated FITC-FSA, suggesting a general downregulation in the LSECs of *Glmp*^*gt/gt*^ mice. This observation was further supported by the results of the qPCR and western blot analyses, which showed a marked reduced expression of *Fcgr2b* mRNA (Fig 5D), and protein (Fig 5E and 5F) in *Glmp*^*gt/gt*^ liver compared to WT.

### LSEC morphology and fenestration in *Glmp*^*gt/gt*^ livers in situ

The structural hallmark of the LSEC is the numerous open fenestrae, which are arranged in sieve plates [2]. Liver fibrosis is correlated with the defenestration of LSECs in several disease models [6]. To examine the morphology of LSECs in *Glmp*^*gt/gt*^ mice, liver samples from 4 months old (n = 4) and 9–10 months old (n = 4) *Glmp*^*gt/gt*^ male mice and WT controls (n = 4, aged 4–6 months) included in the in vivo FITC-FSA distribution study were analysed by scanning EM. The liver samples selected for scanning EM were from mice where the value for “% FITC-fluorescence area per liver tissue area” (Fig 2A) was closest to the median value for this parameter within the respective groups.

**Fig 6:**
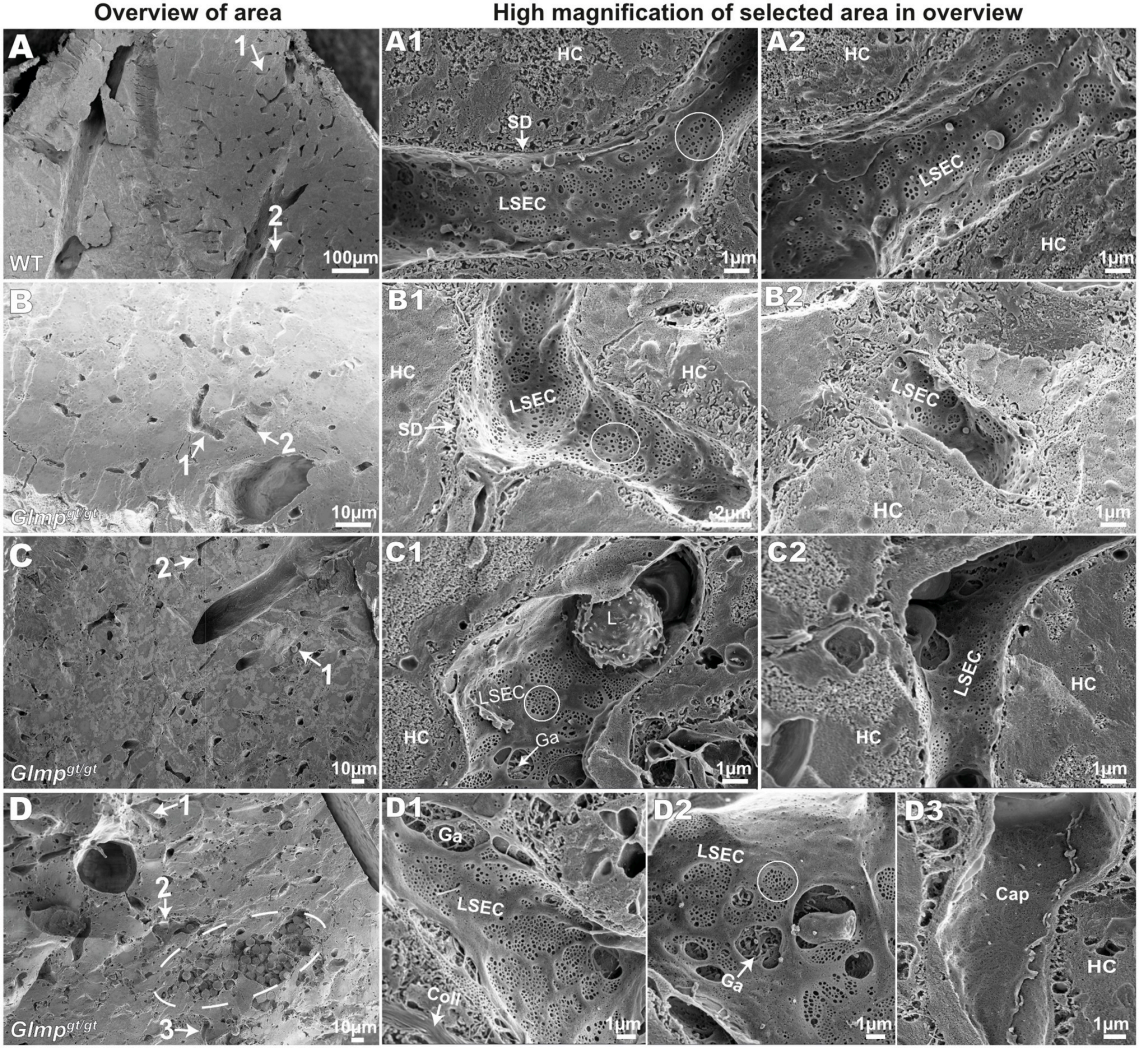
Scanning EM of liver samples from 4 months old WT and *Glmp*^*gt/gt*^ mice. A) Scanning EM image of a liver sample from WT mice. A1 and A2 show high magnification images of two typical sinusoids, labelled 1 and 2 in the overview image in A. B-D) Scanning EM of liver samples from *Glmp*^*gt/gt*^ mice, including overview images and high magnification images from the areas indicated with numbered arrows in the overviews. The LSECs of *Glmp*^*gt/gt*^ were generally well fenestrated, with fenestrae arranged in sieve plates (circles in A1, B1, C1, D2). Large gaps (Ga) were observed in LSECs close to or within areas with hepatocyte damage and infiltration of immune cells (C1, D1-2). D3 shows a non-fenestrated capillary; these were only observed in areas with hepatocyte destruction and infiltration of leukocytes (dashed ellipse in the overview image in D. Abbreviations in A-D: LSEC, liver sinusoidal endothelial cell; HC, hepatocyte; SD, space of Disse; Ga, gap in LSEC; Coll, collagen; Cap, non-fenestrated capillary; L, leukocyte.

The WT livers showed sinusoids with highly fenestrated LSECs (Fig 6A1 and 6A2). The fenestrae were typically arranged in sieve plates and there were few gaps (defined as open holes > 400 nm in diameter) in the cells. LSECs of *Glmp*^*gt/gt*^ mice (Fig 6B–6D) were also well fenestrated, with fenestrae in sieve plates (indicated by circles), including areas with inflammation and fibrosis. However, more gaps were observed in LSECs of the *Glmp*^*gt/gt*^ mice in sinusoids close to, or within areas with hepatocyte damage and infiltration of immune cells (Fig 6C1, 6D1 and 6D2). Capillaries with thick-walled endothelial cells without fenestrations were observed in regions with severe hepatocyte damage, accumulation of inflammatory cells, and destruction of sinusoids. These were judged to represent new vessels (Fig 6D3).

**Fig 7:**
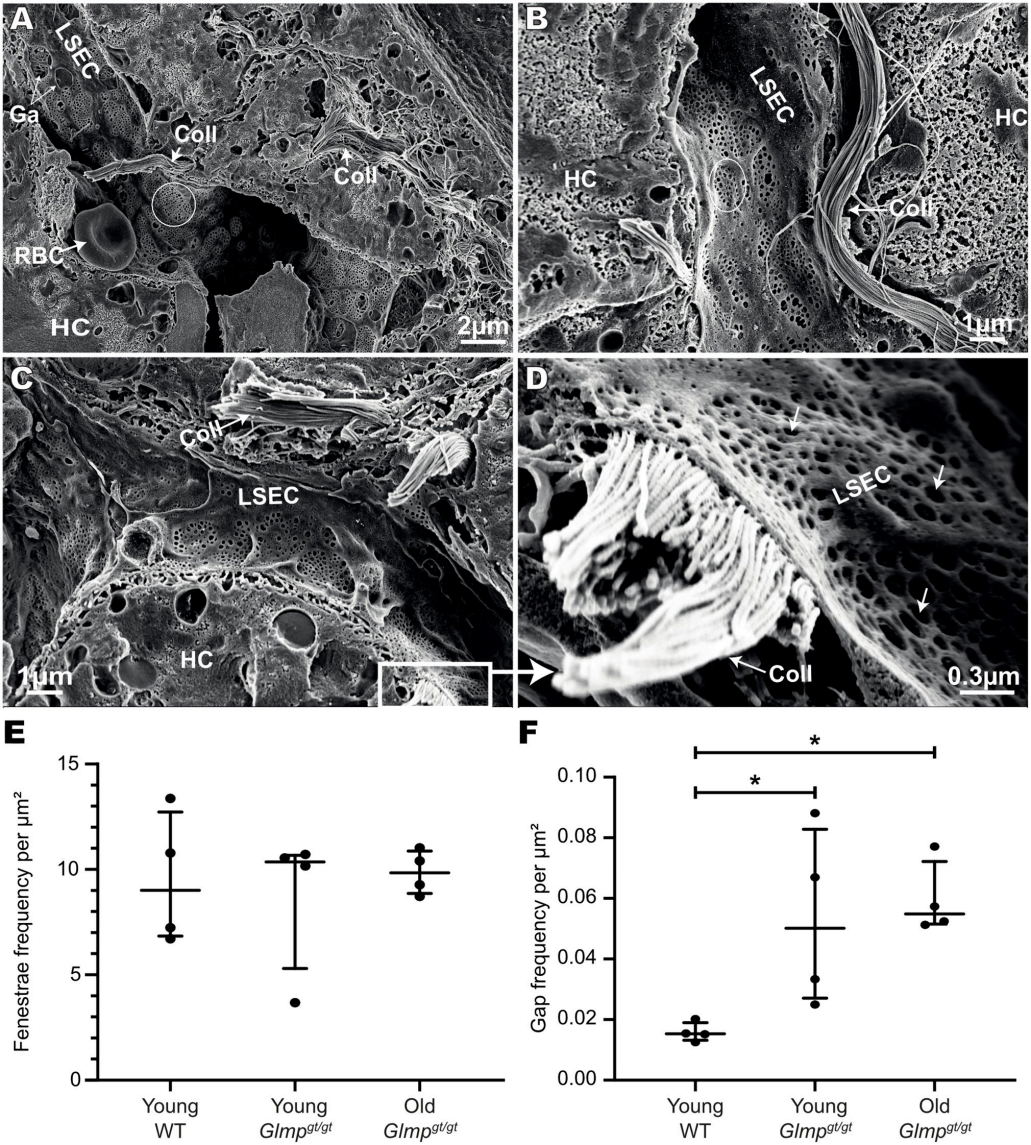
Scanning EM of liver samples from 10 months old *Glmp*^*gt/gt*^ mice, and frequency of fenestrae and gaps in LSECs of *Glmp*^*gt/gt*^ and WT mice in situ. A-C) Scanning EM images of representative sinusoids in the liver of 10 months old *Glmp*^*gt/gt*^ mice. D) Insert in image C, showing part of a highly fenestrated LSEC overlaying a collagen bundle. White circles in A-B show fenestrae arranged in sieve plates, while arrows in D point to single fenestrae in sieve plates. Abbreviations in A-D: LSEC, liver sinusoidal endothelial cell; HC, hepatocyte; RBC, red blood cell; Ga, gap in LSEC; Coll, collagen. E) Frequency of fenestrae (i.e. number of open holes 30–400 nm in diameter, per μm^2)^, and F) frequency of gaps (open holes > 400 nm in diameter, per μμm^2)^ in LSECs were measured on scanning EM images of liver samples from 4 young WT mice (age: 4–6 months), 4 young *Glmp*^*gt/gt*^ mice (age: 4 months), and 4 old *Glmp*^*gt/gt*^ mice (age: 9–10 months). The images were captured at 20.000 x magnification, and 206 images (11–29 images/liver) were analysed as described in Methods. Each dot represents the average value for one mouse, i.e. the value included in the statistical analysis (Kruskal-Wallis test), the median value for each group is presented as a horizontal line, and the upper and lower lines represent the interquartile range. *p-value < 0.05.

Interestingly, in the liver of the 9–10 months old *Glmp*^*gt/gt*^ mice, LSECs were found to be highly fenestrated even in sinusoids with high amount of collagen bundles located in the perisinusoidal space of Disse (Fig 7A–7D). At this age, tissue injury is reported to be less severe than in 4 months old mice [39] but liver collagen content is still significantly higher than in WT mice (Fig 2B).

Quantitative image analysis of SEM images captured at 20,000x magnification showed no significant difference in the fenestrae frequency (defined as number of open holes, 30–400 nm in diameter, per cell area) between liver samples from *Glmp*^*gt/gt*^ and WT mice (Fig 7E). However, compared to WT livers, the *Glmp*^*gt/gt*^ sinusoidal endothelium showed higher frequency of gaps (holes > 400 nm) (Fig 7F). Gap formation was most prominent in the regions with hepatocyte damage and aggregates of inflammatory cells.

### Possible compensatory sites of clearance of blood-borne scavenger receptor ligands in liver fibrosis

**Fig 8:**
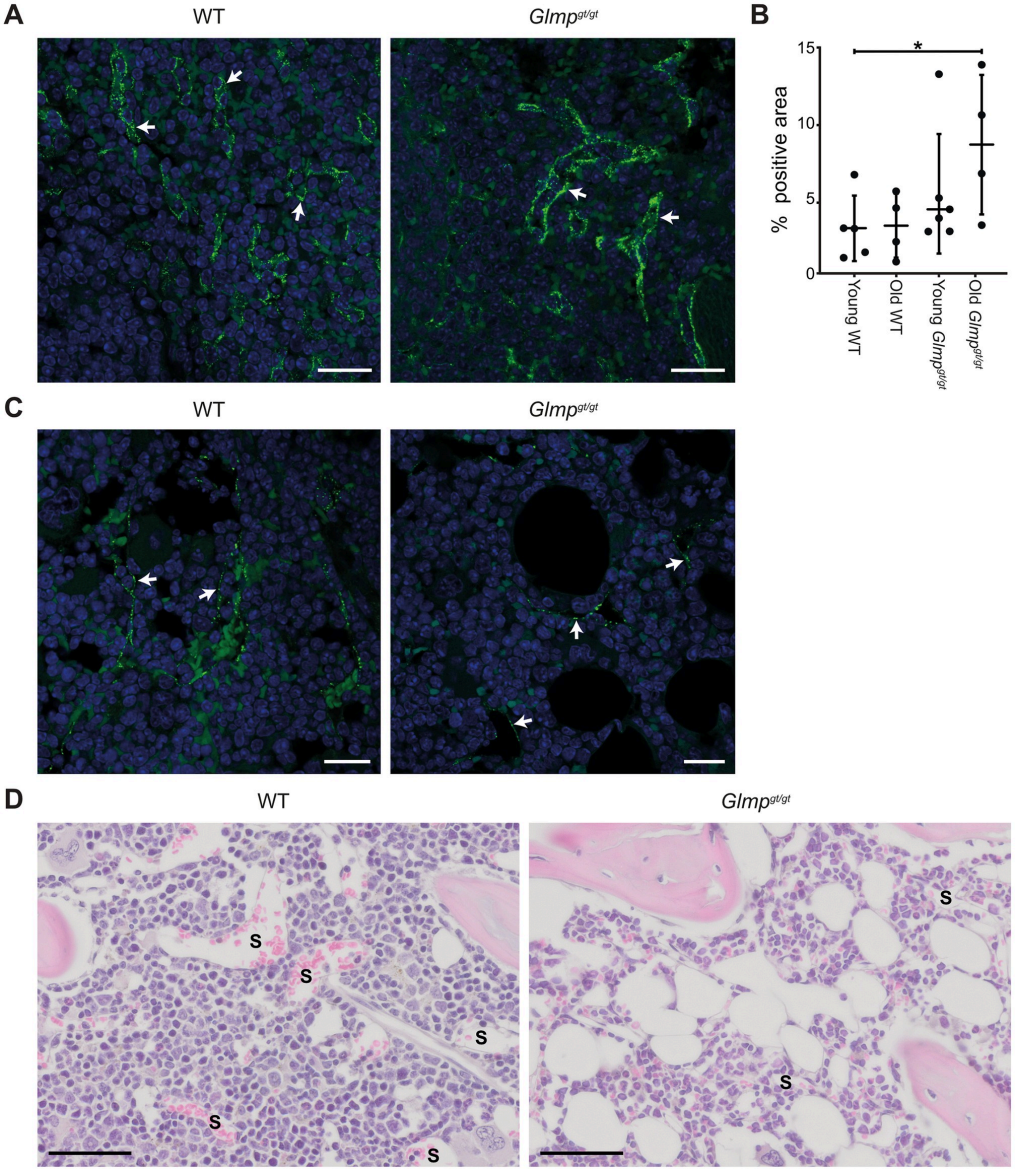
Uptake of FITC-FSA in sinusoids of bone marrow and spleen. Distribution of FITC-FSA uptake in sinusoids of the spleen (A) and bone marrow (C) 10 min after intravenous injection of ligand in *Glmp*^*gt/gt*^ and WT mice. Specific FITC-fluorescence is seen as small, bright green dots (arrows) in the sinusoidal lining cells. Scale bars: 20 μm. B) FITC-FSA uptake in the spleen, presented as % area covered by FITC-fluorescence in tissue sections. Groups: Young WT, 3–6 months (n = 5); old WT, 9–10 months (n = 4); young *Glmp*^*gt/gt*^, 4 months (n = 6); and old *Glmp*^*gt/gt*^, 9–10 months (n = 4). Medians are presented as horizontal lines, and the upper and lower lines represent the interquartile range. Significantly different in old *Glmp*^*gt/gt*^ vs WT, * p-value <0.05, One-way non-parametric ANOVA on ranks (Kruskal-Wallis test). D) Images of bone marrow sections from WT and *Glmp*^*gt/gt*^ mice, stained with haematoxylin and eosin. S, sinusoid. Scale bars: 50 μm.

Although LSECs represent the largest population of scavenger endothelial cells in the mammalian body [13, 54] spleen and bone marrow are also reticuloendothelial organs [55], carrying sinusoidal endothelial cells with high endocytic activity [12, 56]. We found that intravenously administered FITC-FSA was widely distributed in the sinusoids of spleen red pulp, and bone marrow in WT and *Glmp*^*gt/gt*^ mice (Fig 8A and 8C). In both organs, specific fluorescence was observed as small fluorescent dots in the sinusoidal lining cells, revealing uptake in endothelial cells. Image analysis of spleen tissue sections indicated a higher uptake of FITC-FSA in the spleen of *Glmp*^*gt/gt*^ mice compared to WT (significant for the old *Glmp*^*gt/gt*^ group vs. young WT, p< 0.05) (Fig 8B). The distribution of FITC-FSA in bone marrow was not quantified due to difficulties with the method caused by the more irregular structure of the organ.

Notably, the bone marrow of *Glmp*^*gt/gt*^ mice appeared pale at gross inspection while the bone marrow of WT mice was deeply red. Histological examination showed less cellularity and many vacuoles in the *Glmp*^*gt/gt*^ bone marrow, compared to WT animals (Fig 8D). This explains the previous observation that *Glmp*^*gt/gt*^ mice have significantly reduced erythrocyte and lymphocyte numbers in blood compared to WT controls [39].

## Discussion

The LSECs act both as scavenger cells for blood-borne waste macromolecules and colloids, and as sieve cells, allowing the free transfer of lipoproteins and solutes between hepatocytes and blood through their numerous open fenestrae [1]. In this study we examined the in situ LSEC ultrastructure and scavenger function at two different stages in the development of liver pathology in the *Glmp*^*gt/gt*^ mouse, i.e. around 4 months when hepatocyte destruction, oval cell activation, hepatic stellate cell activation, and inflammation are peaking, and at 9–10 months when there is a better balance between injury and repair processes [39]. Liver inflammation and fibrosis are generally associated with capillarization of the sinusoidal endothelium which involves loss of LSEC porosity and formation of a continuous basal lamina underneath the cells [7, 57]. However, preserved LSEC fenestration despite a proinflammatory liver microenvironment has been reported in mice fed on a high-fat diet [58]. By using a method where we cracked open the liver tissue, thus allowing visualization of many sinusoids at the same time by scanning EM, we found that LSECs of the *Glmp*^*gt/gt*^ livers showed well-preserved fenestrae in most sinusoids, including sinusoids with thick collagen bundles located in the space of Disse. Quantitative image analysis showed that while the number of fenestrae were similar in LSECs in *Glmp*^*gt/gt*^ and WT mice, significantly more gaps (defined as holes > 400 nm in diameter) were observed in the LSECs of the *Glmp*^*gt/gt*^ mice. The gaps were larger in the areas with visible hepatocyte damage and accumulation of inflammatory cells. However, even severely damaged LSECs contained some intact sieve plates and were thin-walled cells, while capillaries with thick-walled non-fenestrated endothelial cells, which were observed in areas with focal tissue destruction and many inflammatory cells, likely represent newly formed capillaries.

The focal LSEC damage observed in the *Glmp*^*gt/gt*^ mice may represent a primary injury to the cells, and/or be secondary to hepatocyte injury. A detailed study by Massa Lopez et al. on the lysosomal transporter MFSD1 suggests that LSECs may be especially vulnerable to loss of the ubiquitously expressed GLMP/MFSD1 lysosomal transporter complex [42]. GLMP is a single pass N-glycosylated lysosomal membrane protein [41] and Massa Lopez et al. found that the protein is a critical accessory subunit for MFSD1 and essential for the maintenance of normal MFSD1 levels in lysosomes and vice versa. Germline deletion of *Mfsd1* in mice produced a similar liver phenotype as *Glmp*^*gt/gt*^ mice, with focal liver injury with damage of sinusoids, inflammation and collagen accumulation, new vessel formation, and splenomegaly [42]. The authors further created a conditional mouse model (*Mfsd1*^*flox/flox*^ *Tie2 cre+*) which lacked the expression of *Mfsd1* in endothelial cells and Kupffer cells but not in hepatocytes. This model showed the same liver pathology as the germline *Mfsd1* knockout, suggesting that loss of the GLMP/MFSD1 lysosomal transporter complex has a direct negative effect on LSEC function [42]. The substrate for the transporter is unknown, nor why LSECs are damaged only focally. However, LSECs have a high lysosome content, reflecting their role as highly active scavenger cells [12, 59]. The liver injury in *Glmp*^*gt/gt*^ mice is most severe in the periportal zone of the hepatic lobule, which is the first zone to receive the blood, and, with it, blood-borne tissue turnover products, toxins, and other substances that are endocytosed by the cells. We therefore hypothesize that the heavier scavenging load in the periportal area makes the LSECs in this zone more vulnerable to the loss of a lysosomal transporter for low-molecular-weight catabolic end-products than LSECs located closer to the central vein.

To examine LSEC scavenging in the *Glmp*^*gt/gt*^ mouse we used FSA which is a well-established model ligand used to evaluate endocytic activity in LSECs [1, 60], and compared the in vivo uptake and distribution pattern of FITC-labeled FSA in liver sinusoids of WT and *Glmp*^*gt/gt*^ mice 10 min after intravenous administration. Treatment of albumin with formaldehyde cross-links free amino groups and thus increases the net negative charge of the protein. Such a shift in charge favors the protein uptake via scavenger receptors [61]. Studies in rats and mice have shown that intravenously injected radiolabeled FSA disappears from blood within a few minutes and distributes mainly to liver, where it is cleared by scavenger receptor-mediated endocytosis in LSECs [22, 31, 32]. The injection dose of FITC-labeled FSA (2 μg/g body weight) in the present study was chosen to be high enough to monitor differences in LSEC uptake between genotypes by image analysis on liver sections but low enough to avoid saturation of the uptake system, which will prolong the circulatory half-life. This may allow ligand uptake in cells with a less efficient scavenger function.

The uptake of FITC-FSA was significantly lower in liver sinusoids of *Glmp*^*gt/gt*^ mice compared to WT controls, with almost no ligand uptake in areas with fibrosis. The reduced uptake may be caused by several factors, including reduced liver perfusion and portal hypertension, focal LSEC destruction as demonstrated by scanning EM in areas with inflammatory cell aggregates, and downregulation of LSEC scavenger receptors. The development of splenomegaly in *Glmp*^*gt/gt*^ mice [37, 39] supports the hypothesis of portal hypertension, and decreased blood flow due to damaged sinusoids, focal leukocyte aggregates, and fibrosis are likely to have contributed to the reduced FITC-FSA uptake in LSECs.

The highly efficient scavenger function of LSECs can be ascribed partly to their constitutive expression of several multi-ligand high-affinity endocytosis receptors [12, 13]. The physiological role of scavenger receptors is mainly to remove cellular debris and molecular waste from turnover processes and serve as part of the host defense. The major LSEC receptor for FSA is suggested to be stabilin-2 as over 50% of the uptake of this ligand could be inhibited by an antibody to whole rat stabilin-2 in endocytosis experiments with primary rat LSECs [28]. FSA also binds to stabilin-1 [27] but the relative involvement of this receptor in the LSEC uptake is not known. Stabilin-2 is specifically expressed in LSECs among liver cells both in rodents and humans [15, 17, 18, 62] and is also present in sinusoidal endothelial cells of lymph nodes, spleen and bone marrow [17, 18, 56]. Stabilin-1 shows a similar distribution as stabilin-2 in liver and other organs but is in addition expressed in high endothelial venules in lymph nodes and subpopulations of macrophages in skin and lymph nodes and can be upregulated in response to proinflammatory stimuli [15, 63–67]. The present study found that *Stab1* and *Stab2* expression in liver tissue was similar in age-matched WT and *Glmp*^*gt/gt*^ mice. The reduced liver uptake of FITC-FSA in *Glmp*^*gt/gt*^ mice compared to WT mice could therefore not be explained by differential *Stab1*, or *Stab2* gene expression. Unfortunately, stabilin-2 staining on paraffin embedded tissue in our study was unsuccessful. However, stabilin-1 immune staining of liver of *Glmp*^*gt/gt*^ mice revealed that the receptor was expressed in macrophages in inflammatory cell aggregates, and these were often FITC-FSA negative. At the same time, the expression of stabilin-1 in more normal tissue followed the distribution pattern of FITC-FSA. Upregulation of stabilin-1 has been previously reported at sites of epithelial damage and immune cell recruitment in chronic liver disease [64]. Moreover, the receptor has been reported to function as an atypical adhesion molecule mediating trans-endothelial migration of regulatory T cells, B cells and myeloid cells in liver inflammation [66, 68–71].

We further examined the LSEC expression of the mannose receptor (*Mrc1*) and FcγRIIb (*Fcgr2b*). The mannose receptor is a C-type lectin with roles in immunity and glycoprotein homeostasis [72, 73]. The receptor is widely expressed in LSECs where it mediates endocytosis of a wide range of endogenous and exogenous ligands [13], and expressed to a varying extent in Kupffer cells [12, 20, 53]. The expression of *Mrc1* was almost similar in liver samples from age-matched WT and *Glmp*^*gt/gt*^ mice. Positive mannose receptor staining was observed along the sinusoids, colocalizing with FITC-FSA in a pattern typical for LSECs in both genotypes. In contrast to stabilin-1, the mannose receptor was expressed in relatively few cells at sites of inflammatory cell accumulation in the *Glmp*^*gt/gt*^ livers.

A prominent finding in our study was a significant and widespread loss of one of the LSEC signature receptors, FcγRIIb, in the liver of *Glmp*^*gt/gt*^ mice, both at protein level (measured by immune fluorescence on sections and western blot of whole liver lysates) and mRNA level (measured by qPCR). Notably, the antibody used does not distinguish between FcγRII and -RIII. However, the only Fc receptor expressed in LSECs is the endocytic FcγRIIb2 [16, 21], and the staining pattern in WT mice was uniform along sinusoids and similar to the staining pattern of the SE-1 antibody in rats [20, 74] which is specific for the FcγRIIb in rat LSECs [19]. FcγRIIb2 is a splice variant of FcγRIIb, the only FcγRII in mouse [75, 76]. The liver is the dominating organ expressing FcγRIIb [21, 77] with approximately 90% of the liver receptor pool located in the LSECs [21]. Interestingly, the FcγRIIb expression in *Glmp*^*gt/gt*^ liver was markedly reduced not only in sinusoids in areas with severe pathology but also in regions with sinusoidal endothelial cells that had accumulated FITC-FSA (Fig 5A and 5B), and showed preserved fenestration by scanning EM (Figs 6 and 7). Furthermore, the expression of FcγRIIb was similarly low in *Glmp*^*gt/gt*^ mice at 4 months and 9–10 months of age. However, the level of proinflammatory cytokines and liver collagen production are highest around 4 months [39].

The reduced expression of this receptor in the liver may result from the proinflammatory micro-environment in the *Glmp*^*gt/gt*^ mice; however, a link between loss of GLMP and reduced *Fcgr2b* gene expression, via a hitherto unknown pathway cannot be excluded. Reduced FcγRIIb expression is reported in damaged LSECs in chronic liver inflammation and cirrhosis [12], and single-cell RNA sequencing of liver of mice with CCl_4_ induced liver fibrosis showed loss of *Fcgr2b* in the pericentral zone of the hepatic lobule, i.e. in the zone with most severe pathology [78]. FcγRIIb2 is an inhibitory, endocytic Fc receptor, which is essential for silent removal of small soluble immune complexes from blood [16, 21] and functional studies in rats with CCl_4_-induced liver cirrhosis showed delayed clearance of soluble immune complexes as well as low ligand reactivity in cirrhotic areas [79]. Reduced liver uptake of these ligands enhances the risk of immune complex accumulation in tissues, and FcγRIIb deficient mice show an increased risk of developing systemic lupus erythematosus [80, 81]. Downregulation of FcγRIIb is further reported in non-alcoholic fatty liver disease in humans [82], and, interestingly, the authors also observed a significant negative correlation between FcγRIIb expression in liver tissue and high serum levels of blood lipids, type 4 collagen and hyaluronan in the patients [82]. Hyaluronan is a ligand for stabilin-2 and is cleared from blood mainly by LSECs [28, 83], and enhanced serum level of hyaluronan has been suggested as a functional marker for decreased LSEC endocytosis function in vivo [84].

Despite persistent liver inflammation and fibrosis [37, 39], and impaired scavenging capacity of the liver sinusoidal endothelium (reported in this study), *Glmp*^*gt/gt*^ mice are rather long-lived and show normal behaviour and breeding [39], suggesting that compensatory clearance mechanisms exist for blood-borne macromolecules that are normally eliminated by LSECs. Likewise, human patients often live for many years with liver fibrosis and may show few clinical symptoms suggesting that the liver has a large reserve capacity and/or other organs take over some of the scavenging load. While the LSECs constitute by far the largest reservoir of scavenger endothelial cells in the mammalian body [13, 54], endothelial cells with high endocytic capacity for scavenger receptor ligands are also located in spleen and bone marrow sinusoids [12, 56]. We found that FITC-FSA was widely distributed to sinusoidal endothelial cells of these organs in both *Glmp*^*gt/gt*^ and WT mice. We, therefore, hypothesize that scavenger endothelial cells in these locations may compensate to some extent for decreased LSEC scavenger function in chronic liver disease. The increased spleen size in the *Glmp*^*gt/gt*^ mice (1.5x that of WT [37]) may further lead to a higher uptake in this organ. Interestingly, the bone marrow of *Glmp*^*gt/gt*^ mice showed reduced cellularity of the hematopoietic tissue and more fat than WT mice, explaining the previously reported reduction in blood erythrocytes and leukocyte numbers in these mice [39]. Anemia is a common finding among patients with liver disease, affecting around 70% of patients with liver cirrhosis [85]. However, bone marrow aplasia in the context of liver disease is rare although cases associated with hepatitis are well reported. Viral agents are the most common triggers of hepatitis-associated aplastic anemia, but noninfectious causes are also involved in the pathogenesis [85]. Significantly decreased numbers of hematopoietic stem cells, mesenchymal stem cells, Schwann cells, and neural fibers were reported in bone marrow from patients with advanced liver cirrhosis compared to patients with less severe disease, accompanied with lower hemoglobin and platelet counts [86].

## Conclusion

In this study we report impaired liver clearance capacity, as expressed by reduced LSEC uptake of a soluble scavenger receptor model ligand (FITC-FSA), in the long-lived *Glmp*^*gt/gt*^ mouse model of persisting, multifocal liver injury and fibrosis. The mice further showed extensive downregulation of the FcγRIIb in LSECs. Scanning EM revealed that LSECs in most hepatic sinusoids (i.e. outside the focal areas of tissue injury and inflammatory cell aggregates) in the *Glmp*^*gt/gt*^ mice were intact and showed normal number of fenestrae but increased number of gaps (holes > 400 nm in diameter).

## Supporting information

S1 Fig Test of goat anti-human MMR/CD206 antibody for use in mouse. Figure in A: Western blot of mannose receptor expression in protein lysates of mouse liver sinusoidal endothelial cells (LSEC; mouse strain: C57Bl/6JRj), reduced and non-reduced samples, 30 μg protein loaded per lane. The blot was stained with goat anti-human MMR/CD206 antibody (R&D Systems, Cat. No AF2534) at 1 μg/ml, following the protocol in Methods–Western Blots. Secondary antibody was donkey anti-goat IgG (H+L) cross-absorbed, HRP (Invitrogen, Cat. No A16005, diluted 1:10.000). A strong positive band was observed at approximately 180–200 kDa. The reported size of the mannose receptor in pig and rat LSEC is 180 kDa [25]. Two lower bands in the reduced lane are likely to represent proteolytic cleavage products. B) Confocal laser scanning microscopy images of immune labelled paraffin sections of liver from WT and mannose receptor knock-out mice (MR-KO; C57BL/6 background). The MR-KO mouse model is described in [73], and liver samples were collected from in-house bred mice for the study in [45]. Sections were stained with the goat anti-human MMR/CD206 antibody following the immunohistochemistry protocol in Methods. Positive staining for the mannose receptor is seen as red fluorescence along the liver sinusoids in the WT liver, while no positive staining was observed in the MR-KO liver.
(TIF)

S2 Fig Test of a rabbit anti-human stabilin-1 antibody for use in mouse, and analysis of stabilin-1 expression in WT and *Glmp*^*gt/gt*^ liver. Figure in A: Western blot of protein lysates of mouse liver sinusoidal endothelial cells (LSEC) (mouse strain: C57Bl/6JRj) and mouse stabilin-1 transfected HEK293 cells. Lane 1–3: LSEC (15, 30, 45 μg protein loaded); lane 4: No protein; lane 5: non-transfected HEK293 (30 μg); lane 6: mouse stabilin-1 transfected HEK293 (30 μg), lane 7: HEK293 vector control (30 μg). Staining was performed following the protocol in Methods-Western blot. Primary antibody was rabbit anti-human stabilin-1 (Atlas, Cat. No HPA005434; 1 μg/ml), and secondary antibody was goat anti-rabbit (IgG), HRP (Abcam, Cat. No ab205718, diluted 1:40.000). This produced three bands close to 240 kDa and above, in the LSEC lanes, and 2–3 bands in the same region in the mouse stabilin-1 transfected HEK293 lane. In addition, a lower band was observed in the LSEC samples. Mouse and human stabilin-1 show 81.8% homology [15] and several biochemical species have been described for human stabilin-1 (previously named MS1), including a precursor of 280 kDa, a mature protein of 300 kDa, and two forms of 220 and 120 kDa produced by proteolytic cleavage of the 300 kDa form [15, 65, 87]. B-C) Western blots of liver protein lysates from B) 3 WT mice, and 3 *Glmp*^*gt/gt*^ mice, aged 4 months, and C) 3 WT mice, and 3 *Glmp*^*gt/gt*^ mice, aged 9 months, using the same protocol as in A. Protein loaded per lane: Liver lysates, 25 μg; LSEC, 10 μg; mouse stabilin-1 HEK293 (mSt1-HEK), 7 μg. Beta-actin was used as loading control.
(TIF)

S3 Fig Distribution of FITC-FSA in normal mouse liver. The figure shows confocal laser scanning micrographs of the typical distribution pattern of FITC-FSA uptake (green fluorescence dots) in mouse liver 10 min after intravenous administration of ligand (injection dose: 2 μg/g body weight). Liver macrophages were stained with antibodies to CD68 (red fluorescence in A), or VSIG4 (red fluorescence in B). FITC-FSA was widely distributed in the liver sinusoids in a pattern typical for uptake in sinusoidal endothelial cells. The VSIG4 staining suggests some additional uptake in sinusoidal macrophages (insert in B; and S4 Fig. Z-stack video).
(TIF)

S4 Fig Z-stack video of distribution of FITC-FSA and VSIG4 in WT mouse liver. Z-stack of confocal laser scanning micrographs of the typical distribution pattern of FITC-FSA (green fluorescence dots) in mouse liver sinusoids 10 min after intravenous administration of ligand. Liver macrophages were stained with an antibody to VSIG4 (red fluorescence). Microscope: Zeiss LSM800 equipped with a 40x water objective (NA 1.2).
(AVI)

S1 Table Primers used for real-time quantitative PCR (qPCR). (PDF)

S2 Table Primer validation (qPCR). (PDF)

S1 Raw images The file contains the raw blot images for Figs 4E, 4F, 5E and 5F and S1A, S2A–S2C Figs. (PDF)
