# Supplementary figures and images for: Liver sinusoidal endothelial cells show reduced scavenger function and downregulation of Fc gamma receptor IIb, yet maintain a preserved fenestration in the Glmpgt/gt mouse model of slowly progressing liver fibrosis
Source: PLoS One. 2023 Nov 1;18(11):e0293526. doi: 10.1371/journal.pone.0293526 (PMC10619817; doi:10.1371/journal.pone.0293526)

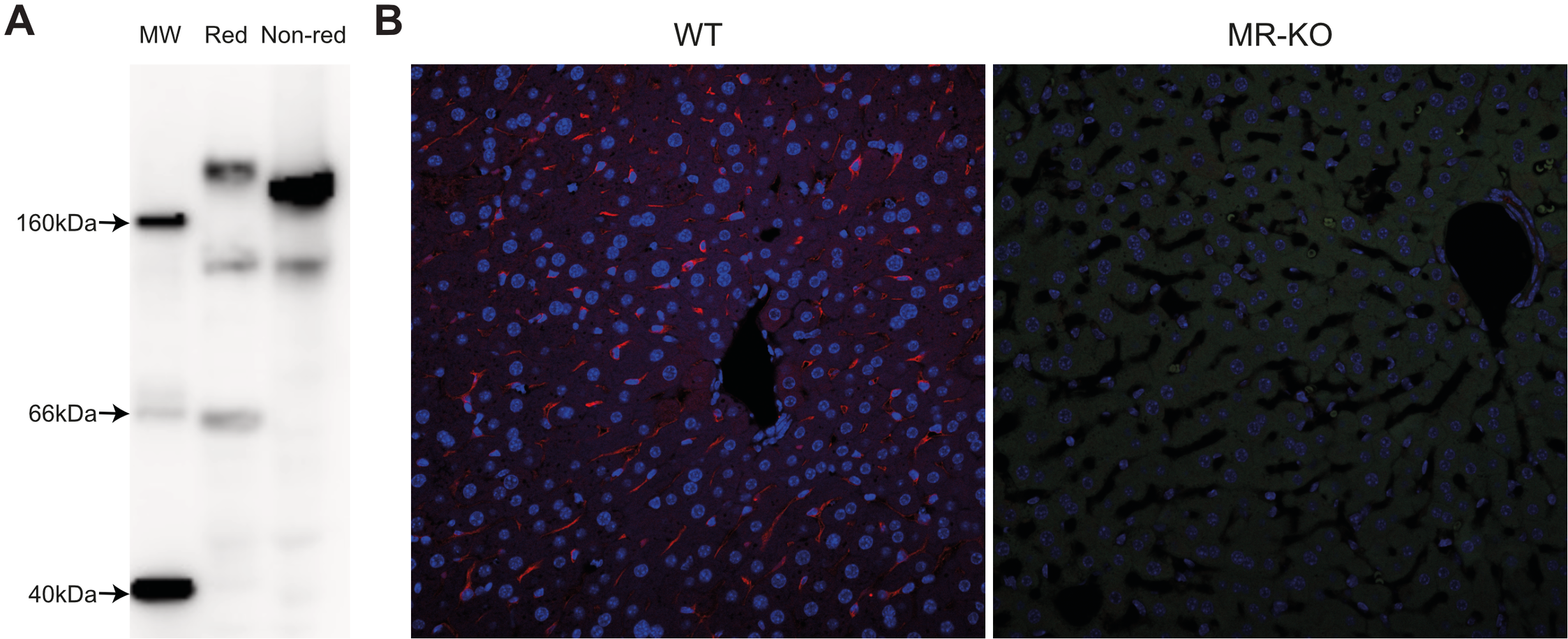

Supplement: S1 Fig — Figure in A: Western blot of mannose receptor expression in protein lysates of mouse liver sinusoidal endothelial cells (LSEC; mouse strain: C57Bl/6JRj), reduced and non-reduced samples, 30 μg protein loaded per lane. The blot was stained with goat anti-human MMR/CD206 antibody (R&D Systems, Cat. No AF2534) at 1 μg/ml, following the protocol in Methods–Western Blots. Secondary antibody was donkey anti-goat IgG (H+L) cross-absorbed, HRP (Invitrogen, Cat. No A16005, diluted 1:10.000). A strong positive band was observed at approximately 180–200 kDa. The reported size of the mannose receptor in pig and rat LSEC is 180 kDa [25]. Two lower bands in the reduced lane are likely to represent proteolytic cleavage products. B) Confocal laser scanning microscopy images of immune labelled paraffin sections of liver from WT and mannose receptor knock-out mice (MR-KO; C57BL/6 background). The MR-KO mouse model is described in [73], and liver samples were collected from in-house bred mice for the study in [45]. Sections were stained with the goat anti-human MMR/CD206 antibody following the immunohistochemistry protocol in Methods. Positive staining for the mannose receptor is seen as red fluorescence along the liver sinusoids in the WT liver, while no positive staining was observed in the MR-KO liver. (TIF) [file pone.0293526.s001.tif]

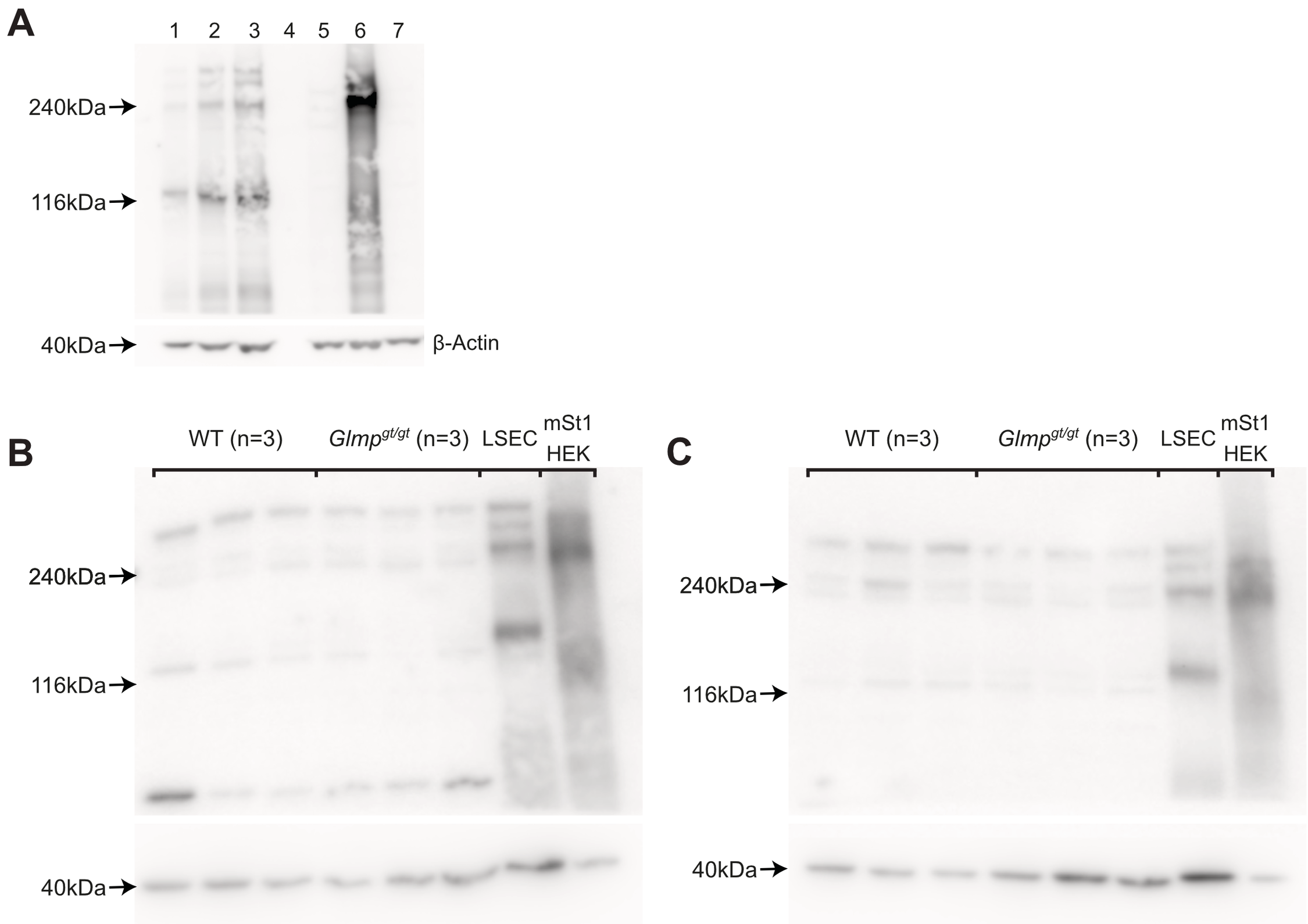

Supplement: S2 Fig — Figure in A: Western blot of protein lysates of mouse liver sinusoidal endothelial cells (LSEC) (mouse strain: C57Bl/6JRj) and mouse stabilin-1 transfected HEK293 cells. Lane 1–3: LSEC (15, 30, 45 μg protein loaded); lane 4: No protein; lane 5: non-transfected HEK293 (30 μg); lane 6: mouse stabilin-1 transfected HEK293 (30 μg), lane 7: HEK293 vector control (30 μg). Staining was performed following the protocol in Methods-Western blot. Primary antibody was rabbit anti-human stabilin-1 (Atlas, Cat. No HPA005434; 1 μg/ml), and secondary antibody was goat anti-rabbit (IgG), HRP (Abcam, Cat. No ab205718, diluted 1:40.000). This produced three bands close to 240 kDa and above, in the LSEC lanes, and 2–3 bands in the same region in the mouse stabilin-1 transfected HEK293 lane. In addition, a lower band was observed in the LSEC samples. Mouse and human stabilin-1 show 81.8% homology [15] and several biochemical species have been described for human stabilin-1 (previously named MS1), including a precursor of 280 kDa, a mature protein of 300 kDa, and two forms of 220 and 120 kDa produced by proteolytic cleavage of the 300 kDa form [15, 65, 87]. B-C) Western blots of liver protein lysates from B) 3 WT mice, and 3 Glmpgt/gt mice, aged 4 months, and C) 3 WT mice, and 3 Glmpgt/gt mice, aged 9 months, using the same protocol as in A. Protein loaded per lane: Liver lysates, 25 μg; LSEC, 10 μg; mouse stabilin-1 HEK293 (mSt1-HEK), 7 μg. Beta-actin was used as loading control. (TIF) [file pone.0293526.s002.tif]

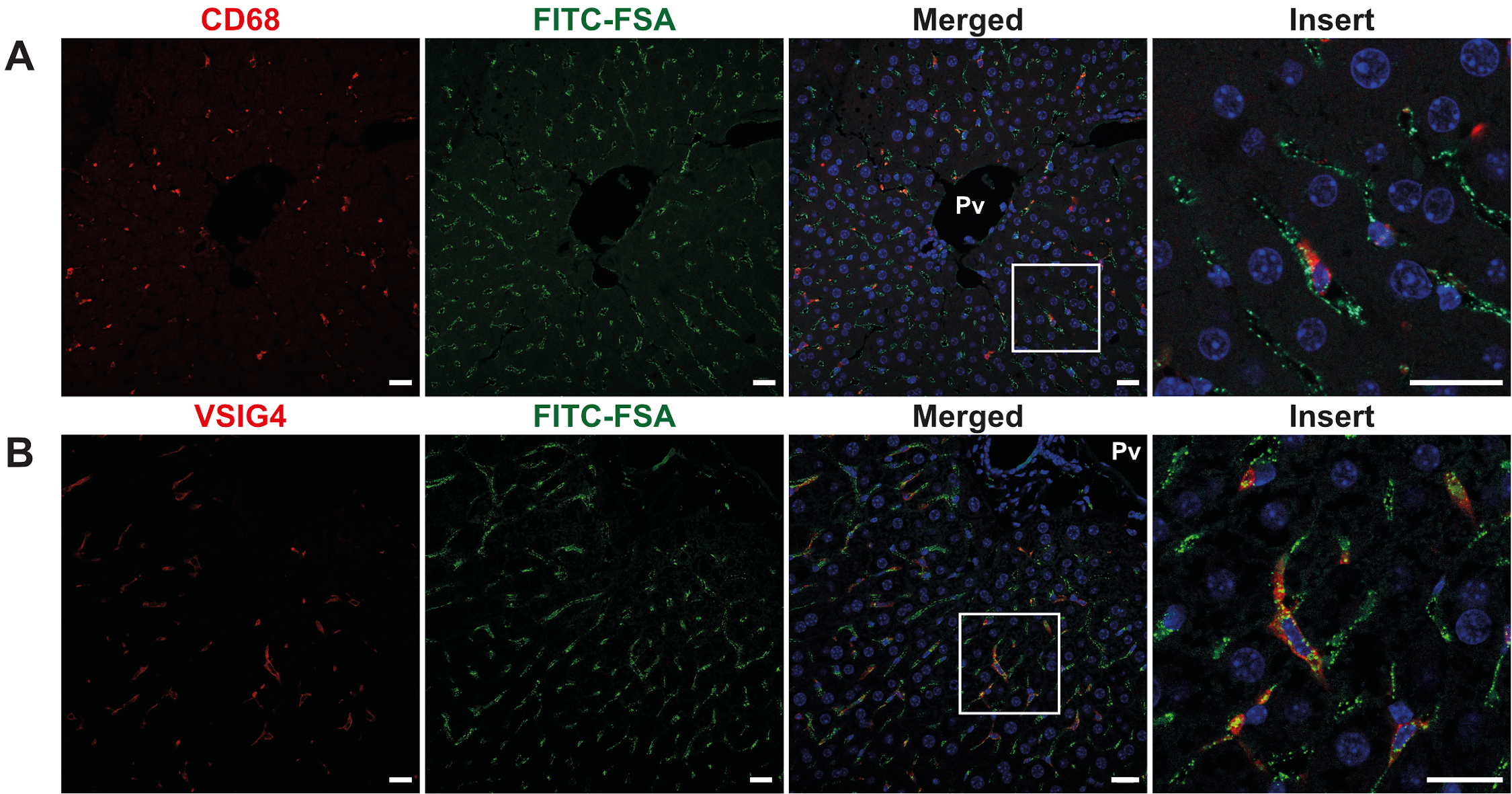

Supplement: S3 Fig — The figure shows confocal laser scanning micrographs of the typical distribution pattern of FITC-FSA uptake (green fluorescence dots) in mouse liver 10 min after intravenous administration of ligand (injection dose: 2 μg/g body weight). Liver macrophages were stained with antibodies to CD68 (red fluorescence in A), or VSIG4 (red fluorescence in B). FITC-FSA was widely distributed in the liver sinusoids in a pattern typical for uptake in sinusoidal endothelial cells. The VSIG4 staining suggests some additional uptake in sinusoidal macrophages (insert in B; and S4 Fig. Z-stack video). (TIF) [file pone.0293526.s003.tif]
