# Supplementary material for: Liver sinusoidal endothelial cells show reduced scavenger function and downregulation of Fc gamma receptor IIb, yet maintain a preserved fenestration in the Glmpgt/gt mouse model of slowly progressing liver fibrosis
Source: PLoS One. 2023 Nov 1;18(11):e0293526. doi: 10.1371/journal.pone.0293526 (PMC10619817; doi:10.1371/journal.pone.0293526)
Supplement: S1 Table — (PDF) [file pone.0293526.s005.pdf]

S1 Table. Primers used for real-time quantitative PCR (qPCR)

**Symbol**	**Accession number**	Primers	Sequence	Length	Amplicon	Range
Stab2	NM_138673	**St2-f1**	ACCTACAACCAGCTCTCCTAT	21	113	6770-6790
		**St2-r1**	GCACATTTCTTAGAGGCGTAGA	22		6882-6861
Stab1	NM_138672	**St1-f1**	GCTGTTGCCCTGGTCACTAT	20	112	6022-6041
		**St1-r1**	CACTGCCCACTTCCACGTAT	20		6133-6114
Mrc1	NM_008625	**MR-f1**	TTCCATCGAGACTGCTGCTG	20	168	3909-3928
		**MR-r1**	CCAGAGGGATCGCCTGTTTT	20		4076-4057
Fcgr2b	NM_001077189	**FC-f1**	TGATCTGGAAGAAGCTGCCAA	21	73	977-997
		**FC-r1**	GGCTTCGGGATGCTTGAGAA	20		1049-1030
