# Supplementary material for: Liver sinusoidal endothelial cells show reduced scavenger function and downregulation of Fc gamma receptor IIb, yet maintain a preserved fenestration in the Glmpgt/gt mouse model of slowly progressing liver fibrosis
Source: PLoS One. 2023 Nov 1;18(11):e0293526. doi: 10.1371/journal.pone.0293526 (PMC10619817; doi:10.1371/journal.pone.0293526)
Supplement: S2 Table — (PDF) [file pone.0293526.s006.pdf]

S2 Table. Primer validation (qPCR)

Primer Name	Primer concentration	Standard curve				
		Range (copies/well)	Slope	y-intercept	Efficiency	R2
St2-f1<br>St2-r1	400nM F, 400 nM R	10E6-10	-3.3594	34.78	1.98	1
St1-f1<br>St1-r1	400 nM F, 200 nM R	10E6-10	-3.364	34.23	1.99	1
MR-f1<br>MR-r1	400nM F, 200 nM R	10E6-10	-3.3303	35.41	2	1
FC-f1<br>FC-r1	600nM F, 600 nM R	10E6-10	-3.3022	35.9	2.01	1
