# Supplementary material for: Liver sinusoidal endothelial cells show reduced scavenger function and downregulation of Fc gamma receptor IIb, yet maintain a preserved fenestration in the Glmpgt/gt mouse model of slowly progressing liver fibrosis
Source: PLoS One. 2023 Nov 1;18(11):e0293526. doi: 10.1371/journal.pone.0293526 (PMC10619817; doi:10.1371/journal.pone.0293526)
Supplement: S1 Raw images — (PDF) [file pone.0293526.s007.pdf]

Mannose Receptor, raw data for Figures 4E and 4F: Coomassie blue stain membranes (top images) and the same membranes immunostained for MR and β-actin (bottom images)

Black lines indicate the cutting of the membranes.

Fig. 4E

Fig.4F

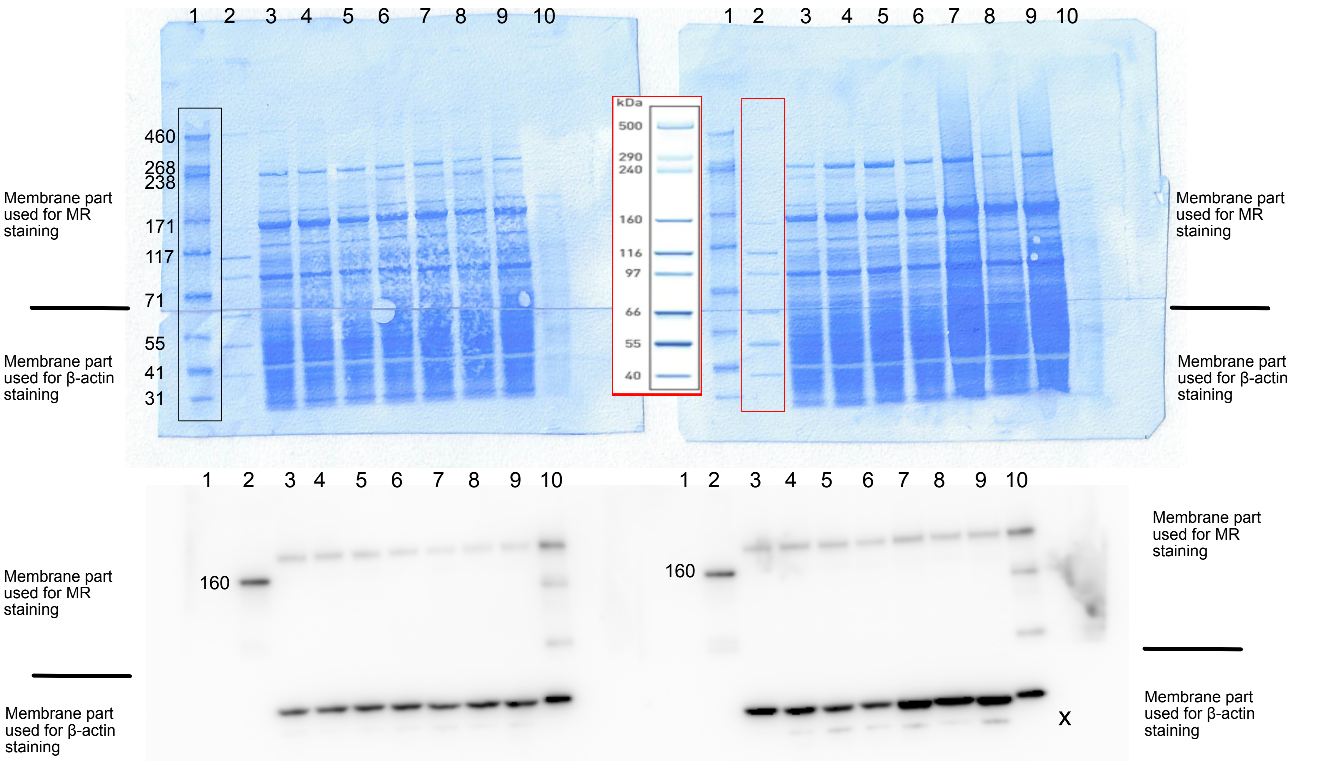

Sample loading Fig. 4E:

1. Himark Prestained ladder (band sizes in black)
2. Himark Unstained protein standard (band sizes in insert in red box)
3. WT 4 mo - 25 µg
4. WT 4 mo - 25 µg
5. WT 4 mo - 25 µg
6. WT 4 mo - 25 µg
7. $Glmp^{gt/gt}$ 4 mo - 25 µg
8. $Glmp^{gt/gt}$ 4 mo - 25 µg
9. $Glmp^{gt/gt}$ 4 mo - 25 µg
10. mLSEC-5 µg

Sample loading Fig. 4F:

1. Himark Prestained ladder (band sizes in black)
2. Himark Unstained protein standard (band sizes in insert in red box)
3. WT 9 mo - 25 µg
4. WT 9 mo - 25 µg
5. WT 9 mo - 25 µg
6. WT 9 mo - 25 µg
7. $Glmp^{gt/gt}$ 9 mo - 25 µg
8. $Glmp^{gt/gt}$ 9 mo - 25 µg
9. $Glmp^{gt/gt}$ 9 mo - 25 µg
10. mLSEC-5 µg

Membranes were developed for 30 seconds using ImageQuant™ LAS 4000.

Raw data for Figures 5E and 5F: Membranes were immunostained with primary antibody against FcγRII/III (CD16/CD32) (top images), and β-actin (bottom images)

Fig. 5E

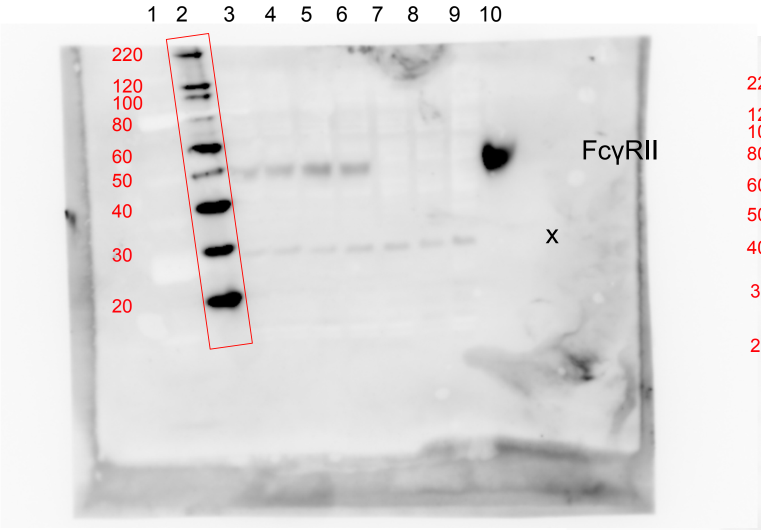

Fig. 5F

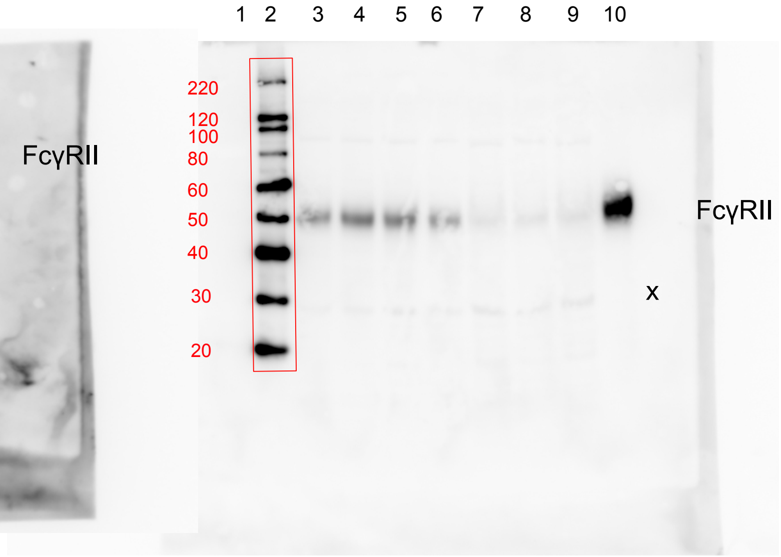

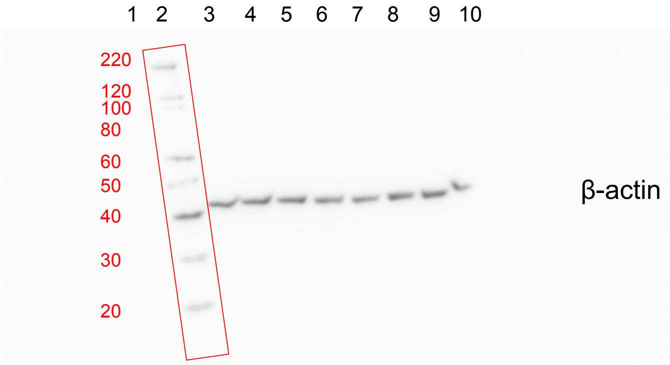

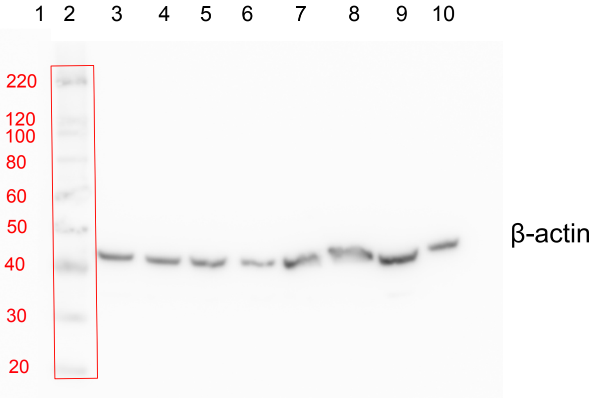

Sample loading Fig. 5E:

1. Precision Plus Dual color standard (not visible)
2. MagicMark standard protein ladder (band sizes in red)
3. WT 4 mo - 25 µg
4. WT 4 mo - 25 µg
5. WT 4 mo - 25 µg
6. WT 4 mo - 25 µg
7. Glmp$^{gt/gt}$ 4 mo - 25 µg
8. Glmp$^{gt/gt}$ 4 mo- 25 µg
9. Glmp$^{gt/gt}$ 4 mo- 25 µg
10. mLSEC-5 µg

Sample loading Fig. 5F:

1. Precision Plus Dual color standard (not visible)
2. MagicMark standard protein ladder (band sizes in red)
3. WT 9 mo - 25 µg
4. WT 9 mo- 25 µg
5. WT 9 mo- 25 µg
6. WT 9 mo- 25 µg
7. Glmp$^{gt/gt}$ 9 mo- 25 µg
8. Glmp$^{gt/gt}$ 9 mo- 25 µg
9. Glmp$^{gt/gt}$ 9 mo- 25 µg
10. mLSEC-5 µg

Development time (ImageQuant™ LAS 4000):

- FcγRII/III: 120 seconds
- β-actin: 80 seconds

Mannose Receptor raw data for S1 Fig.A :Coomassie blue stain membranes (image on the left) and the same membrane immunostained for MR (image on the right)

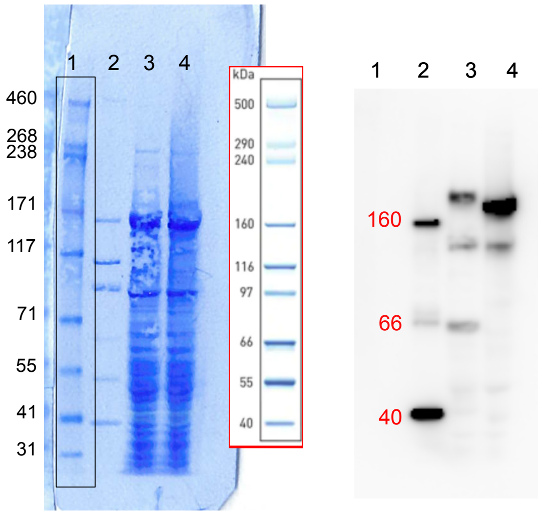

Sample loading S1 Fig.A:

1. Himark Prestained ladder (band sizes in black)
2. Himark Unstained protein standard (band sizes in insert in red box and in red)
3. mLSEC reduced- 30 µg
4. mLSEC non-reduced- 30 µg

Membranes were developed for 20 seconds using ImageQuant™ LAS 4000.

Raw data for S2 Fig.A : Coomassie blue stain membrane (image on the left) and the same membrane immunostained for Stabilin-1 and β-actin (image on the right)

Black lines indicate the cutting of the membrane.

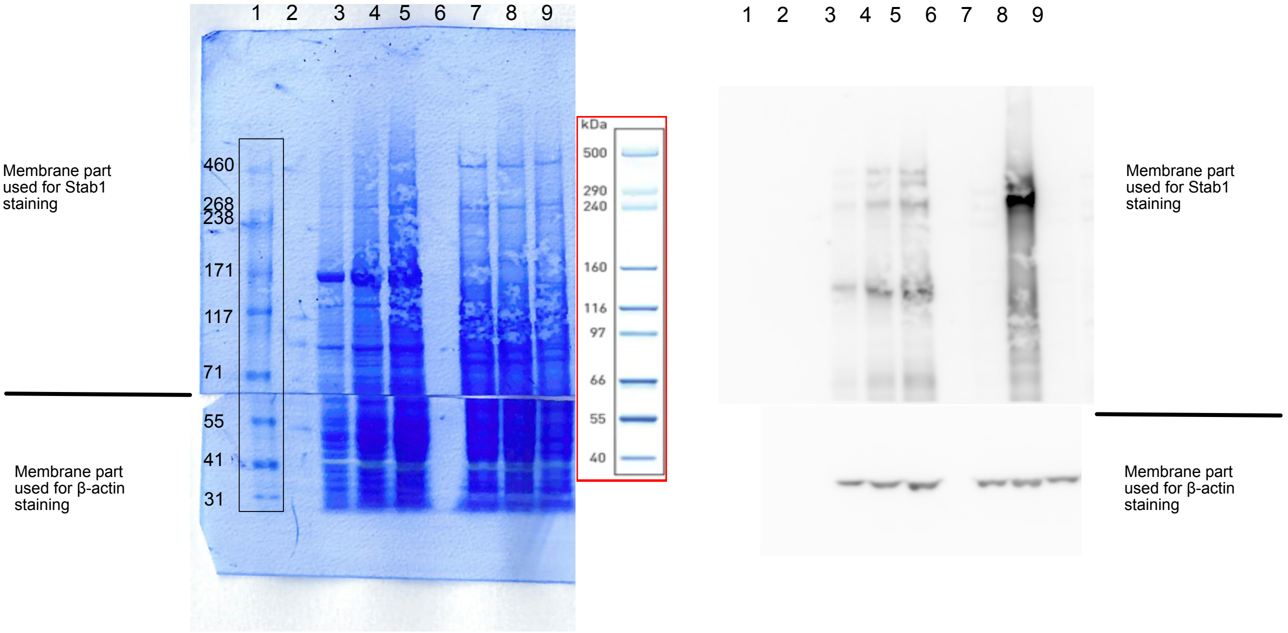

Sample loading S2 Fig.A :

1. Himark Prestained ladder (band sizes in black)
2. Himark Unstained protein standard (band sizes in insert in red box)
3. mLSEC- 15 µg
4. mLSEC- 30 µg
5. mLSEC- 45 µg
6. Empty well
7. HEK293 cells- 30 µg
8. Mouse Stabilin1 HEK293 - 30 µg
9. Emtpy vector HEK293- 30 µg

Development time (ImageQuant™ LAS 4000):

- Stab1: 6 minutes
- β-actin: 1 minute

Stabilin-1 raw data for S2 Fig. B and C: Coomassie blue stain membranes (top images) and the same membranes immunostained for Stabilin-1and β-actin (bottom images)

Black lines indicate the cutting of the membranes.

S2 Fig. B

S2 Fig. C

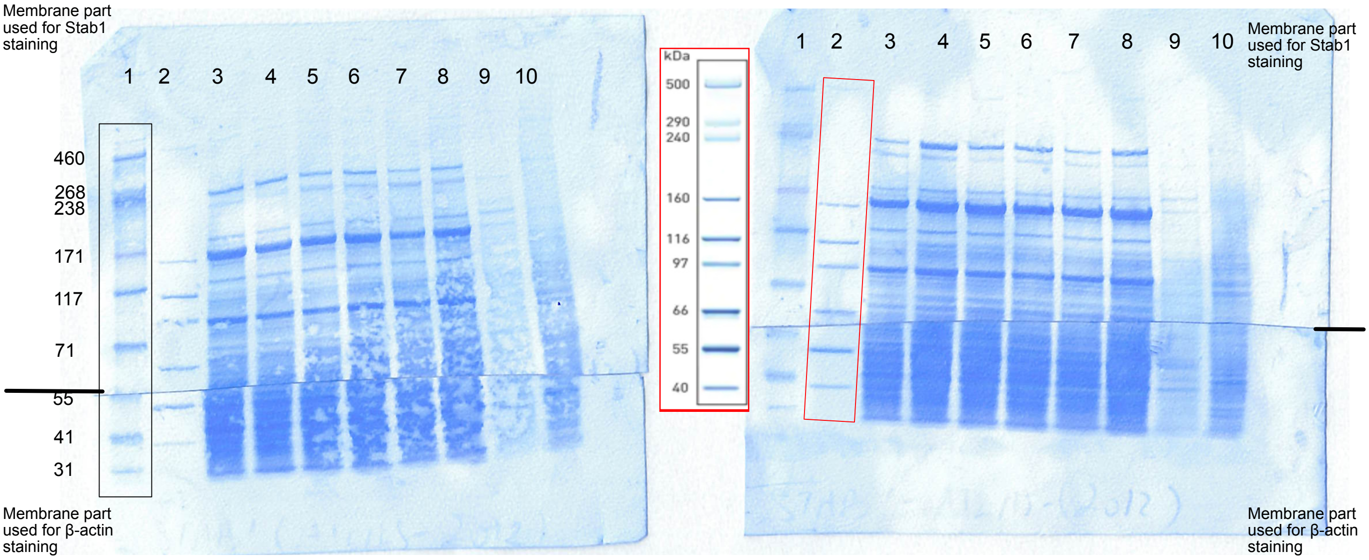

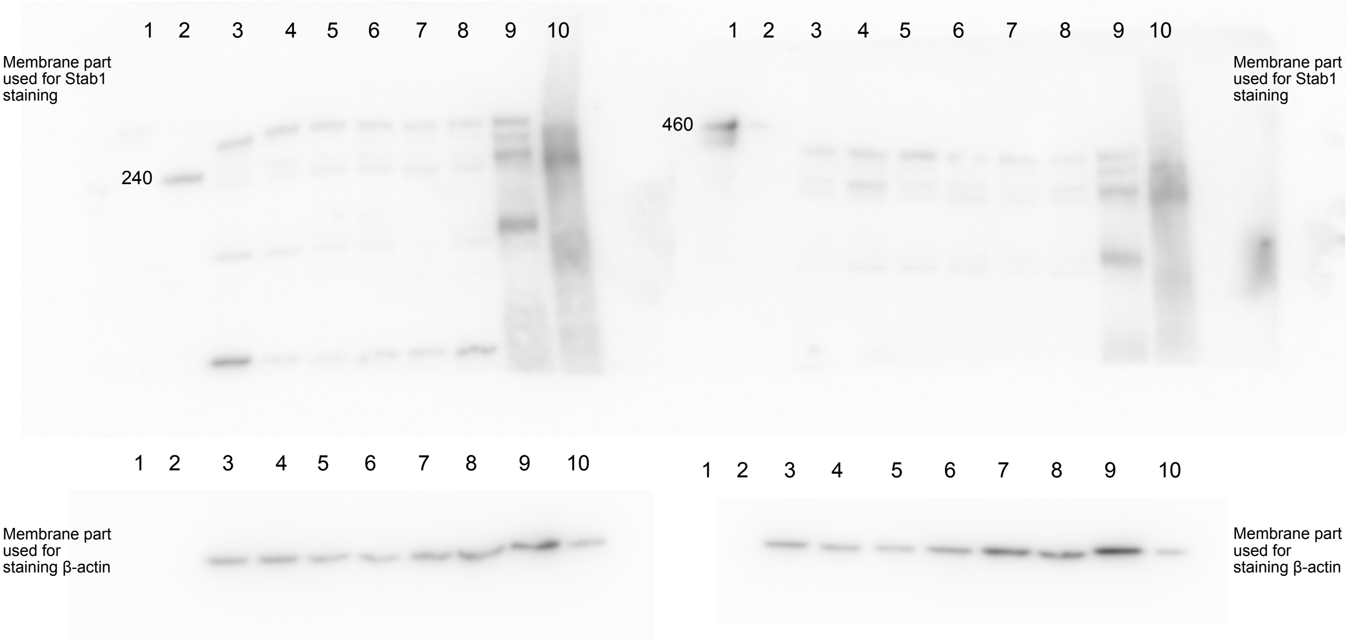

Sample loading S2 Fig. B:

1. Himark Prestained ladder (band sizes in black)
2. Himark Unstained protein standard (band sizes in insert in red box)
3. WT 4 mo
4. WT 4 mo
5. WT 4 mo
6. Glmp$^{gt/gt}$ 4 mo
7. Glmp$^{gt/gt}$ 4 mo
8. Glmp$^{gt/gt}$ 4 mo
9. mLSEC-10 µg
10. mouse Stabilin1 HEK293 - 7µg

Sample loading S2 Fig. C:

1. Himark Prestained ladder (band sizes in black)
2. Himark Unstained protein standard (band sizes in insert in red box)
3. WT 9 mo
4. WT 9 mo
5. WT 9 mo
6. Glmp$^{gt/gt}$ 9 mo
7. Glmp$^{gt/gt}$ 9 mo
8. Glmp$^{gt/gt}$ 9 mo
9. mLSEC-10 µg
10. mouse Stabilin1 HEK293- 7µg

Development time (ImageQuant™ LAS 4000):

- Stab1: 6 minutes
- β-actin: 1 minute
